# All-cause mortality following internal fixation for isolated fractures in non-polytrauma admissions: a comprehensive 10-year nationwide linked data retrospective cohort study

**DOI:** 10.1136/bmjopen-2026-122170

**Published:** 2026-09-08

**Authors:** Walter Muruet, Caroline Hing, Paul Aylin, Alex Bottle, Omar Musbahi

**Affiliations:** 1 Dr Foster Unit, Department of Primary Care and Public Health, School of Public Health, Imperial College London, London, UK; 2 St George’s University of London, London, UK

**Keywords:** Fractures, Bone, ORTHOPAEDIC & TRAUMA SURGERY, EPIDEMIOLOGY, Mortality

## Abstract

**Abstract:**

**Introduction:**

Skeletal fractures are a leading cause of death and disability and a growing global public health concern. Internal fixation is a common surgical treatment indicated for several fractures, yet there are few comprehensive epidemiological studies describing mortality following the procedure.

**Methods:**

Data were extracted from an England-wide administrative database (Hospital Episode Statistics, HES) and linked to mortality records from the Office for National Statistics (ONS). All records from patients aged 18 years or older who were admitted due to a non-polytrauma single fracture and underwent internal fixation between 2013 and 2023 were included. Crude and adjusted mortality rates were calculated as well as the restricted mean time lost.

**Results:**

A total of 630 758 admissions were included in the primary analysis. Of these, 55% were female, and the mean±SD age was 59±23 years. Primary open reduction internal fixation was the most common procedure (54% of cases), followed by closed reduction internal fixation (35%). Overall, mortality rates following internal fixation were 2% within 30 days, 5% within 90 days and 10% within 365 days. Mortality rates were described by fracture region and reduction procedure. Variation in mortality rates across fracture regions and procedures was attenuated in the case-mix-adjusted estimates.

**Conclusions:**

This study described national crude and adjusted mortality rates following internal fixation for the treatment of skeletal fractures. These findings provide population-level descriptive benchmarks for clinical and epidemiological interpretation and may inform decision-making by clinicians and patients as well as the design of randomised controlled trials and other research studies.

**STRENGTHS AND LIMITATIONS OF THIS STUDY**

- This study used a large, nationally representative cohort that included 630 758 admissions over 10 years, with virtually complete coverage of National Health Service (NHS) hospital admissions and internal fixation procedures in England.
- Linkage to the Office for National Statistics mortality records enabled a virtually complete assessment of short-term and long-term all-cause mortality.
- This study provides comprehensive outcome reporting, evaluating mortality across multiple time horizons, fracture regions and fixation procedures using crude, case-mix-adjusted and restricted mean time lost estimates.
- As a retrospective cohort study using administrative data, this study is liable to misclassification due to the reliance on administrative coding. This may have introduced errors in fracture, comorbidity and procedure classification as well as limited granularity regarding procedures.
- Some important clinical factors, such as physiological status, fracture morphology, implant characteristics and intraoperative details, were unavailable. Therefore, there is the possibility of residual confounding in the case-mix-adjusted estimates.

## Introduction

### Background

 Bone fractures are a leading cause of death and disability^1^ and a growing global public health concern. In 2019 alone, there were an estimated 178 million new fractures worldwide, with 455 million prevalent cases of acute and long-term fracture sequelae. These represented a 33% and 70% increase, respectively, from 1990 to 2019.^2^ National epidemiological studies further support an increase in bone fracture incidence in Western countries,^3^ particularly for vertebral,^4^ clavicular,^5^ humeral,^5–8^ hand,^5^ pelvic,^5^ femoral,^5 9^ tibial and foot and ankle^5^ fractures. In England, the annual incidence of fractures has been estimated at approximately 3.6 per 100 person-years,^10^ with more than 1.8 million fractures treated by the National Health Service (NHS) each year.^11^ Furthermore, although there is some disagreement,^12^ most projections anticipate an increase in the incidence of fractures in the coming years due to demographic changes.^13 14^ High-quality research into prevention and management is critical as bone fractures are associated with significant morbidity, mortality and healthcare costs.^15–17^

The approach to fracture management encompasses non-operative and surgical methods.^18^ Of the latter, fracture immobilisation can be achieved by combining reduction procedures with internal fixation. Fracture reduction can be closed or open. Closed reduction involves the manual manipulation of the fracture, typically performed in an operating theatre under anaesthesia, whereas open reduction requires the exposure of the bone. Internal fixation involves the surgical placement of implants to hold bone fragments in position until they are united. Commonly used implants for internal fixation can be divided into major categories: wires, pins and screws, plates and intramedullary nails or rods.^18^ The aim is to stabilise the fractured bone, thereby enabling faster healing, early mobility and rehabilitation and, ideally, full functional recovery.^18^

Internal fixation is indicated for a wide array of fractures, including shoulder girdle,^19^ humeral,^20 21^ forearm,^22 23^ pelvic,^24^ femoral^25–27^ and ankle^28^ fractures. However, despite its ubiquity, there is a scarcity of comprehensive epidemiological studies that describe the associated mortality, particularly for non-femoral fractures. Most information is inferred from clinical trials and observational studies focusing on patient subgroups (eg, patients over 70 years old), which significantly hinders their generalisability. As the burden of bone fractures increases worldwide, it is becoming increasingly important to comprehensively describe postoperative mortality to better inform the decisions of clinicians, patients, researchers and policymakers.

In this study, we describe short-term and long-term all-cause mortality following internal fixation for non-polytrauma patients with isolated fractures, regardless of aetiology, stratified by fracture region and fracture reduction method, using a nationwide administrative dataset linked with official mortality records.

### Objectives

The primary aim of this study was to calculate crude mortality rates within 30 days, 90 days and 1 year for all non-polytrauma adult admissions undergoing fracture internal fixation, overall and stratified by reduction method and anatomical fracture region. Secondary aims were (1) to estimate mortality rates adjusted by sociodemographic, clinical and surgical factors and (2) to estimate the restricted mean time lost (RMTL) to death overall for each reduction method and fracture region up to 5 years postprocedure.

## Methods

### Study design and setting

An analysis of inpatient statistics using NHS Hospital Episode Statistics (HES) data was performed. HES collects data on all admissions to NHS hospitals. Because virtually all unplanned hospital activity in England is publicly funded by the NHS,^29^ HES provides comprehensive information on all patients admitted for skeletal fractures across England.

HES records are organised in episodes and spells. An episode represents the period during which a patient is under the care of a given consultant. A spell is composed of one or more episodes and represents a continuous period of time that a given patient spends within a hospital trust.^30^ However, because the management of a health event may require a patient to be transferred to a different trust, temporary adjacent spells were linked into ‘superspells’.^31^ For this study, a hospital admission is defined as a ‘superspell’.

HES includes detailed sociodemographic, clinical and administrative data for each hospital admission. Information is extracted from clinical records by specially trained clinical coders located at each hospital. Sociodemographic information includes age, phenotypic sex, self-reported ethnic group and area of usual residence (urban vs rural). Clinical information includes diagnoses and surgical procedures. One primary and up to 19 secondary diagnoses are recorded using the International Classification of Diseases, 10th revision (ICD-10) codes.^32^ Similarly, one primary and up to 23 secondary procedures are recorded using the Office of Population Censuses and Surveys (OPCS) classification of surgical operations V.4 (OPCS-4).^33^ Administrative information includes dates for admissions, discharges and operations as well as the admission method (ie, elective or emergency), discharge destination and other relevant details. Using these types of data, it is possible to provide a rich description of a patient’s hospitalisation.

### Participants

Data on all admissions occurring between the 1 January 2013 and the 31 January 2023, inclusive, of patients 18 years of age or older, and where the primary OPCS-4 code belonged to a prespecified list of operative procedures, which included primary open reduction and intramedullary fixation (W19.x), primary open reduction and extramedullary fixation (W20.x), other primary open reduction (W22.x), secondary open reduction (W23.x), closed reduction with internal fixation (W24.x) and other internal fixation of bone (W28.x) were extracted from HES. Secondary open reduction classification included patients who had previously undergone reduction of the same fracture (eg, closed reduction and application of a Plaster of Paris, POP, cast), while other internal fixation of bone refers to internal fixation procedures not classified elsewhere.^33^ A complete list of included OPCS-4 codes, along with their corresponding definitions, is provided in online supplemental table A. Fracture aetiology was not used as an eligibility criterion because the study aimed to provide nationally representative descriptive estimates for all admissions involving an isolated fracture treated with internal fixation. Polytrauma admissions were excluded as well as those with bone fractures in multiple anatomical regions or where the site of fracture was not recorded.

### Variables

Sociodemographic and administrative data were extracted from HES using the Admitted Patient Care (APC) technical output specification as a reference.^34^ Comorbidities were derived from the recorded ICD-10 codes (online supplemental table B). For descriptive statistics purposes, the disease burden was summarised using the Elixhauser index score, aggregated into a single number, with van Walraven’s weights.^35 36^ Elderly patients represent an important subpopulation; therefore, the Hospital Frailty Risk Score^37^ was calculated to identify likely frail patients. The abbreviated injury scale and the injury severity score (ISS)^38^ were used to describe injury severity and were derived from ICD-10 codes using the ICDPIC-R package.^39 40^ OPCS-4 and ICD-10 codes were used to identify the site of fracture (online supplemental table C). The date of death and cause of death were obtained by linkage to the Office for National Statistics (ONS) mortality records. Causes of death were classified into six groups: cardiovascular, infection, respiratory, malignancy, surgical complications and any other cause of death. These groups were based on those reported by Liem *et al*.^41^ However, we also included surgical complications as a separate category (online supplemental table D).

### Statistical analysis

Patient characteristics, including age, phenotypic sex, ethnic group, area of usual residence, admission method, injury severity, comorbidity and frailty burden, time from admission to surgical procedure, length of postprocedure hospital stay and discharge destination, were extracted and summarised using descriptive statistics. Numerical variables were summarised depending on their distribution.

Categorical variables were reported as counts with percentages. Due to the large sample size, even minor differences were likely to achieve statistical significance at or below the 0.05 level; therefore, only those differences that were deemed clinically meaningful and those with a moderate or large effect size were reported. For categorical variables, this was defined as a Cohen’s ω ≥0.30,^42^ while for numerical variables, an η^2^ value≥0.06.^43^

Crude and adjusted mortality rates within 30, 90 and 365 days postprocedure, stratified by reduction method and anatomical region of the fracture, were calculated. For crude rates, CIs were estimated using Wilson’s score method.^44^ Adjusted proportions were estimated using marginal standardisation. These were calculated using a logistic regression model that adjusted for characteristics previously reported to be associated with mortality after orthopaedic procedures.^45^ These included sociodemographic characteristics (age,^46^ sex,^46^ ethnicity,^47 48^ area of usual residence), disease burden (individual Elixhauser comorbidities^46 49^ plus osteoporosis,^50^ Paget’s disease, dementia^51^ and delirium,^46^ smoking/nicotine dependence^49^), hospital frailty risk score,^52^ derived ISS,^40^ type of admission (emergency or elective), previous procedures^53^ and year of surgery. CIs for adjusted case fatality rates were estimated using non-parametric bootstrapping.^54^

Survival was described using the RMTL.^55^ RMTL provides a clinically meaningful measure of survival time that is robust to the presence of non-proportional hazards, can be calculated even if less than 50% of participants experienced an event, and represents the average amount of time lost because of death within a specific period. RMTL was calculated as the area above the estimated survival curve from the date of procedure to predefined time horizons. The 1-year, 3-year and 5-year horizons were selected a priori based on clinical input to describe intermediate-term and long-term mortality burden. Five years was chosen as the longest horizon because it provided a clinically meaningful period while retaining adequate follow-up among the included cases.

All analyses were performed using the R software V.4.4.1.^56^

### Missing data and lost to follow-up

The analysis was restricted to complete cases only because the overall percentage of observations with missing data was less than 3%^57^ (online supplemental table E). ONS data provide a virtually complete record of deaths occurring in the country.^58^ Patients without a recorded date of death were assumed to be alive and were censored to 5 February 2024, the latest date of death recorded in the extract.

### Sensitivity analysis

To assess the sensitivity of the mortality estimates to the inclusion of patients with malignancy or other neoplastic disease, a sensitivity analysis excluding patients with evidence of neoplastic processes was performed.

### Patient and public involvement

This research was done without patient involvement. As an observational study using national-level administrative data, it was not possible to involve patients or members of the public.

## Results

### Participants and follow-up

A total of 630 758 admissions (607 721 unique patients) were included in the primary analysis (tables 1 and 2, online supplemental table F). Median follow-up time was 4 years and 250 days, with a maximum of 11 years and 9 days. Pathological fractures, including osteoporotic, malignancy-associated and unspecified, accounted for less than 2% of all included admissions. Malignancy-associated pathological fractures specifically accounted for less than 1% of all admissions. There were 168 524 postprocedure deaths, of which 15 361 (9% of the total) occurred within 30 days, 33 391 (20%) within 90 days, 64 443 (38%) within 365 days and 142 952 (85%) by 5 years. For those deaths, the reported cause of death was malignancy (18%), cardiovascular (11%), infection (5%), respiratory (5%) and any other cause of death (61%). Less than 0.1% were due to surgical complications.

**Table 1:** Cohort characteristics

	Overall	Primary open reduction	Secondary open reduction	Closed reduction	Other internal fixation
Admissions	630 758	339 383	55 322	222 047	14 006
Female, n (%)	346 726 (55.0)	172 643 (50.9)	33 626 (60.8)	133 947 (60.3)	6510 (46.5)
Age in years, mean±SD	58.5±23.0	52.9±21.5	52.8±18.4	68.9±22.8	53.7±19.8
Age group, n (%)					
<45	196 306 (31.1)	130 528 (38.5)	18 441 (33.3)	42 669 (19.2)	4668 (33.3)
45–64	154 255 (24.5)	97 992 (28.9)	20 203 (36.5)	31 461 (14.2)	4599 (32.8)
65+	280 197 (44.4)	110 863 (32.7)	16 678 (30.1)	147 917 (66.6)	4739 (33.8)
Ethnicity, n (%)					
White	525 395 (83.3)	278 527 (82.1)	45 803 (82.8)	189 458 (85.3)	11 607 (82.9)
Black	7131 (1.1)	4560 (1.3)	691 (1.2)	1676 (0.8)	204 (1.5)
Asian	16 806 (2.7)	9881 (2.9)	1460 (2.6)	5104 (2.3)	361 (2.6)
Mixed	3747 (0.6)	2394 (0.7)	366 (0.7)	900 (0.4)	87 (0.6)
Other	11 232 (1.8)	6663 (2.0)	1140 (2.1)	3215 (1.4)	214 (1.5)
Unknown	66 447 (10.5)	37 358 (11.0)	5862 (10.6)	21 694 (9.8)	1533 (10.9)
Area of residence, n (%)					
Urban	489 746 (78.4)	263 444 (78.5)	43 193 (78.8)	172 480 (78.4)	10 629 (76.6)
Town	61 933 (9.9)	32 575 (9.7)	5274 (9.6)	22 613 (10.3)	1471 (10.6)
Rural	72 679 (11.6)	39 651 (11.8)	6338 (11.6)	24 906 (11.3)	1784 (12.8)
Emergency admission, n (%)	415 174 (65.8)	201 023 (59.2)	24 048 (43.5)	185 247 (83.4)	4856 (34.7)
Transport accident, n (%)	52 763 (8.4)	37 643 (11.1)	3354 (6.1)	11 519 (5.2)	247 (1.8)
Dementia, n (%)	62 874 (10.0)	15 746 (4.6)	689 (1.2)	46 147 (20.8)	292 (2.1)
Delirium, n (%)	19 181 (3.0)	4909 (1.4)	209 (0.4)	13 969 (6.3)	94 (0.7)
Smoking/nicotine dependence, n (%)	17 246 (2.7)	9227 (2.7)	1845 (3.3)	5746 (2.6)	428 (3.1)
Alcohol misuse, n (%)	28 859 (4.6)	15 471 (4.6)	2441 (4.4)	10 610 (4.8)	337 (2.4)
Drug abuse, n (%)	7558 (1.2)	4667 (1.4)	744 (1.3)	2001 (0.9)	146 (1.0)
Osteoporosis, n (%)	53 795 (8.5)	18 923 (5.6)	2626 (4.7)	31 717 (14.3)	529 (3.8)
Paget’s disease, n (%)	640 (0.1)	230 (0.1)	25 (0.0)	371 (0.2)	14 (0.1)
Congestive heart failure, n (%)	24 248 (3.8)	7461 (2.2)	519 (0.9)	16 040 (7.2)	228 (1.6)
Cardiac dysrhythmias, n (%)	63 758 (10.1)	21 762 (6.4)	2088 (3.8)	39 123 (17.6)	785 (5.6)
Valvular disease, n (%)	19 256 (3.1)	6150 (1.8)	550 (1.0)	12 348 (5.6)	208 (1.5)
Pulmonary circulation disorders, n (%)	3664 (0.6)	1284 (0.4)	87 (0.2)	2211 (1.0)	82 (0.6)
Peripheral vascular disease, n (%)	10 389 (1.6)	3242 (1.0)	315 (0.6)	6645 (3.0)	187 (1.3)
Systemic arterial hypertension, n (%)	172 989 (27.4)	73 049 (21.5)	11 010 (19.9)	85 737 (38.6)	3193 (22.8)
Paralysis, n (%)	4022 (0.6)	1449 (0.4)	157 (0.3)	2336 (1.1)	80 (0.6)
Neurodegenerative disorders, n (%)	26 205 (4.2)	11 007 (3.2)	1540 (2.8)	13 297 (6.0)	361 (2.6)
Chronic pulmonary disease, n (%)	91 903 (14.6)	42 756 (12.6)	7355 (13.3)	39 809 (17.9)	1983 (14.2)
Diabetes mellitus, n (%)	57 947 (9.2)	24 891 (7.3)	3162 (5.7)	28 706 (12.9)	1188 (8.5)
Hypothyroidism, n (%)	36 816 (5.8)	16 009 (4.7)	2841 (5.1)	17 344 (7.8)	622 (4.4)
Chronic kidney disease, n (%)	40 614 (6.4)	12 435 (3.7)	1007 (1.8)	26 656 (12.0)	516 (3.7)
Liver disease, n (%)	7369 (1.2)	3279 (1.0)	463 (0.8)	3478 (1.6)	149 (1.1)
Peptic ulcer disease, n (%)	1246 (0.2)	481 (0.1)	64 (0.1)	686 (0.3)	15 (0.1)
AIDS/HIV, n (%)	148 (0.0)	95 (0.0)	11 (0.0)	38 (0.0)	4 (0.0)
Lymphoma, n (%)	3104 (0.5)	1146 (0.3)	82 (0.1)	1361 (0.6)	515 (3.7)
Metastatic cancer, n (%)	11 559 (1.8)	3733 (1.1)	148 (0.3)	5068 (2.3)	2610 (18.6)
Solid tumour, n (%)	16 511 (2.6)	5359 (1.6)	316 (0.6)	8904 (4.0)	1932 (13.8)
Connective tissue disease, n (%)	19 023 (3.0)	8631 (2.5)	1341 (2.4)	8618 (3.9)	433 (3.1)
Coagulopathy, n (%)	3198 (0.5)	1299 (0.4)	169 (0.3)	1658 (0.7)	72 (0.5)
Obesity, n (%)	23 617 (3.7)	14 029 (4.1)	3002 (5.4)	5650 (2.5)	936 (6.7)
Weight loss, n (%)	2286 (0.4)	492 (0.1)	28 (0.1)	1754 (0.8)	12 (0.1)
Fluid and electrolyte disorders, n (%)	28 143 (4.5)	8274 (2.4)	563 (1.0)	19 034 (8.6)	272 (1.9)
Blood loss anaemia, n (%)	657 (0.1)	261 (0.1)	19 (0.0)	371 (0.2)	6 (0.0)
Deficiency anaemia, n (%)	12 072 (1.9)	3853 (1.1)	312 (0.6)	7760 (3.5)	147 (1.0)
Psychosis, n (%)	3617 (0.6)	1785 (0.5)	241 (0.4)	1551 (0.7)	40 (0.3)
Depression, n (%)	44 930 (7.1)	23 075 (6.8)	4426 (8.0)	16 500 (7.4)	929 (6.6)
Elixhauser comorbidity index, median (IQR)	0 (0, 3)	0 (0, 0)	0 (0, 0)	0 (0, 6)	0 (0, 7)
Hospital frailty score, median (IQR)	2 (0, 6)	2 (0, 4)	1 (0, 2)	5 (2, 11)	0 (0, 2)
Abbreviated injury score (highest), median (IQR)	0 (0, 0)	0 (0, 0)	0 (0, 0)	0 (0, 0)	0 (0, 0)
Injury severity score, median (IQR)	0 (0, 0)	0 (0, 0)	0 (0, 0)	0 (0, 0)	0 (0, 0)
Injury severity score classification, n (%)					
<9	624 291 (99.0)	337 075 (99.3)	55 162 (99.7)	218 087 (98.2)	13 967 (99.7)
9–15	5457 (0.9)	1828 (0.5)	118 (0.2)	3486 (1.6)	25 (0.2)
16–25	943 (0.1)	450 (0.1)	38 (0.1)	441 (0.2)	14 (0.1)
26+	67 (0.0)	30 (0.0)	4 (0.0)	33 (0.0)	0 (0.0)
Admission to procedure in days, median (IQR)	0 (0, 1)	0 (0, 1)	0 (0, 0)	1 (0, 1)	0 (0, 1)
Previous fixation procedures, median (IQR)	0 (0, 0)	0 (0, 0)	0 (0, 0)	0 (0, 0)	0 (0, 0)
Previous fixation procedures (cat), n (%)					
0	599 291 (95.0)	327 321 (96.4)	48 037 (86.8)	212 701 (95.8)	11 232 (80.2)
1	29 368 (4.7)	11 304 (3.3)	6671 (12.1)	8892 (4.0)	2501 (17.9)
2	1841 (0.3)	677 (0.2)	524 (0.9)	411 (0.2)	229 (1.6)
3	202 (0.0)	72 (0.0)	68 (0.1)	36 (0.0)	26 (0.2)
4+	56 (0.0)	9 (0.0)	22 (0.0)	7 (0.0)	18 (0.1)
Postprocedure stay in days, median (IQR)	1 (0, 10)	1 (0, 4)	1 (0, 1)	8 (1, 19)	1 (0, 4)
Postprocedure stay>28 days, n (%)	53 835 (8.5)	17 385 (5.1)	870 (1.6)	35 162 (15.9)	418 (3.0)
Discharge destination, n (%)					
Home	558 110 (88.5)	316 281 (93.2)	53 995 (97.6)	174 468 (78.6)	13 366 (95.4)
Temporary	15 215 (2.4)	5483 (1.6)	402 (0.7)	9182 (4.1)	148 (1.1)
Care home	27 178 (4.3)	8169 (2.4)	422 (0.8)	18 415 (8.3)	172 (1.2)
Other	12 498 (2.0)	4271 (1.3)	261 (0.5)	7824 (3.5)	142 (1.0)
Death	14 621 (2.3)	4010 (1.2)	143 (0.3)	10 322 (4.6)	146 (1.0)
Unknown	3136 (0.5)	1169 (0.3)	99 (0.2)	1836 (0.8)	32 (0.2)

**Table 2:** Fracture regions by procedure

	Overall	Primary open reduction, internal fixation (primary ORIF)	Secondary open reduction, internal fixation (secondary ORIF)	Closed reduction, internal fixation	Other internal fixation
Admissions	630 758	339 383	55 322	222 047	14 006
Fracture site, n (%)					
Shoulder girdle	31 553 (5.0)	29 465 (8.7)	1343 (2.4)	325 (0.1)	420 (3.0)
Humerus	43 205 (6.8)	36 041 (10.6)	3746 (6.8)	2674 (1.2)	744 (5.3)
Radius	108 690 (17.2)	61 831 (18.2)	29 718 (53.7)	16 808 (7.6)	333 (2.4)
Ulna	24 576 (3.9)	22 099 (6.5)	1597 (2.9)	595 (0.3)	285 (2.0)
Hand	94 327 (15.0)	48 174 (14.2)	4788 (8.7)	38 221 (17.2)	3144 (22.4)
Arm (other)	632 (0.1)	347 (0.1)	61 (0.1)	102 (0.0)	122 (0.9)
Pelvis	2295 (0.4)	1708 (0.5)	139 (0.3)	315 (0.1)	133 (0.9)
Femur	217 706 (34.5)	59 590 (17.6)	3249 (5.9)	149 834 (67.5)	5033 (35.9)
Tibia	47 733 (7.6)	32 253 (9.5)	4104 (7.4)	10 096 (4.5)	1280 (9.1)
Fibula	27 212 (4.3)	21 450 (6.3)	4963 (9.0)	565 (0.3)	234 (1.7)
Leg (other)	17 203 (2.7)	15 822 (4.7)	793 (1.4)	280 (0.1)	308 (2.2)
Tarsus	6344 (1.0)	4645 (1.4)	458 (0.8)	654 (0.3)	587 (4.2)
Foot	8539 (1.4)	5521 (1.6)	338 (0.6)	1524 (0.7)	1156 (8.3)
Other	743 (0.1)	437 (0.1)	25 (0.0)	54 (0.0)	227 (1.6)

ORIF, open reduction internal fixation.

### Distribution of procedures and fracture regions

There was a strong association between procedure and fracture region (ω=0.649). Primary open reduction internal fixation (pORIF) was used to treat a relatively higher number of humeral (11%), shoulder girdle (9%) and ulnar fractures (7%), while around half (54%) of fractures treated with secondary open reduction internal fixation (sORIF) were radial fractures. Only a small proportion of pORIF and sORIF procedures were for femoral fractures (18% and 6%, respectively). Sixty-seven per cent of closed reduction internal fixation (CRIF) interventions were for femoral fractures, with few fractures of the radius (8%), humerus (1%), shoulder girdle (0%), fibula (0%) and ulna (0%) treated by this method.

### Mortality following internal fixation

Overall crude postprocedure mortality rates were 2% within 30 days, 5% within 90 days and 10% within 365 days. Femoral fractures accounted for approximately 90% of all observed deaths within 1 year, humeral fractures for 4%, tibial fractures for 2% and radial fractures for 1% (table 3).

**Table 3:** Crude and adjusted mortality rates by fracture region

Fracture region	Admissions	Within 30 days		Within 90 days		Within 365 days		
		Crude	Adjusted*	Crude	Adjusted*	Crude	Adjusted*	
Shoulder girdle	31 553	0.02% (0.01, 0.05)	0.26% (0.25, 0.26)	0.10% (0.07, 0.14)	1.36% (1.35, 1.37)	0.42% (0.36, 0.50)	5.11% (5.08, 5.13)	
Humerus	43 205	0.92% (0.83, 1.01)	1.88% (1.87, 1.89)	2.68% (2.54, 2.84)	4.87% (4.85, 4.89)	6.20% (5.98, 6.43)	9.81% (9.76, 9.85)	
Radius	108 690	0.07% (0.05, 0.09)	0.38% (0.38, 0.38)	0.21% (0.19, 0.24)	1.33% (1.32, 1.34)	0.88% (0.83, 0.94)	4.81% (4.78, 4.83)	
Ulna	24 576	0.20% (0.15, 0.26)	0.70% (0.70, 0.70)	0.52% (0.44, 0.62)	1.99% (1.98, 2.00)	1.81% (1.65, 1.99)	6.08% (6.05, 6.11)	
Hand	94 327	0.02% (0.01, 0.03)	0.24% (0.23, 0.24)	0.07% (0.06, 0.09)	1.12% (1.12, 1.13)	0.30% (0.26, 0.33)	4.30% (4.27, 4.32)	
Arm (other)	632	0.16% (0.03, 0.89)	0.90% (0.89, 0.91)	0.32% (0.09, 1.15)	1.93% (1.92, 1.95)	1.27% (0.64, 2.48)	6.34% (6.31, 6.37)	
Pelvis	2295	0.78% (0.50, 1.24)	1.27% (1.27, 1.28)	2.14%(1.62, 2.81)	3.71%(3.69, 3.73)	4.97% (4.15, 5.93)	8.28% (8.25, 8.32)	
Femur	217 706	6.68% (6.58, 6.79)	2.72% (2.70, 2.73)	14.31% (14.16, 14.46)	5.83% (5.80, 5.85)	26.76% (26.57, 26.95)	11.20% (11.16, 11.25)	
Tibia	47 733	0.35% (0.30, 0.41)	1.37% (1.37, 1.38)	0.82% (0.74, 0.90)	3.36% (3.35, 3.38)	2.09% (1.96, 2.22)	7.93% (7.89, 7.97)	
Fibula	27 212	0.11% (0.08, 0.16)	0.68% (0.67, 0.68)	0.26% (0.21, 0.33)	1.83% (1.82, 1.84)	0.75% (0.66, 0.86)	5.04% (5.02, 5.07)	
Leg (other)	17 203	0.19% (0.13, 0.26)	0.64% (0.64, 0.65)	0.45% (0.36, 0.57)	1.82% (1.80, 1.83)	1.46% (1.30, 1.66)	5.54% (5.52, 5.57)	
Tarsus	6344	0.03% (0.01, 0.11)	0.25% (0.25, 0.25)	0.11% (0.05, 0.23)	1.06% (1.05, 1.07)	0.58% (0.42, 0.80)	4.94% (4.91, 4.96)	
Foot	8539	0.12% (0.06, 0.22)	0.91% (0.90, 0.91)	0.19% (0.12, 0.30)	1.67% (1.66, 1.68)	0.70% (0.55, 0.90)	5.54% (5.51, 5.56)	
Other	743	0.40% (0.14, 1.18)	1.33% (1.32, 1.34)	1.08% (0.55, 2.11)	3.42% (3.40, 3.44)	2.56% (1.64, 3.96)	6.85% (6.81, 6.88)	

* Adjusted for age, sex, ethnic group, residence setting, comorbidities, hospital frailty risk score, type of admission, number of previous fixation procedures and year of surgery.

pORIF crude mortality rates were 1% within 30 days, 3% within 90 days and 5% within 365 days. When stratified by fracture region, mortality rates for admissions undergoing pORIF for femoral fractures were 6% within 30 days, 13% within 90 days and 24% within 365 days; for humeral fractures, 1% within 30 days, 2% within 90 days and 5% within 365 days; for tibial fractures, 0% within 30 days, 1% within 90 days and 2% within 365 days; and for radial fractures, 0% within 30 days, 0% within 90 days and 1% within 365 days (table 4 *and* online supplemental table I). At 1, 3 and 5 years postprocedure, those undergoing pORIF lost 13, 71 and 164 days to death, respectively (table 5).

**Table 4:** Crude and adjusted mortality rates by procedure

Procedure	Fracture region	Admissions	Within 30 days		Within 90 days		Within 365 days	
			Crude	Adjusted*	Crude	Adjusted*	Crude	Adjusted*
Primary open reduction	Overall	339 383	1.22% (1.18, 1.26)	2.43% (2.42, 2.45)	2.64% (2.59, 2.70)	5.17% (5.14, 5.19)	5.34% (5.27, 5.42)	9.94% (9.90, 9.99)
	Shoulder girdle	29 465	0.02% (0.01, 0.05)	0.25% (0.25, 0.26)	0.10% (0.07, 0.14)	1.32% (1.31, 1.32)	0.41% (0.34, 0.49)	4.90% (4.87, 4.92)
	Humerus	36 041	0.72% (0.64, 0.82)	1.87% (1.86, 1.88)	2.05% (1.91, 2.20)	4.74% (4.71, 4.76)	4.88% (4.66, 5.10)	9.48% (9.45, 9.52)
	Radius	61 831	0.07% (0.05, 0.09)	0.38% (0.37, 0.38)	0.21% (0.18, 0.25)	1.28% (1.27, 1.29)	0.78% (0.71, 0.85)	4.61% (4.59, 4.64)
	Ulna	22 099	0.20% (0.15, 0.27)	0.70% (0.69, 0.70)	0.52% (0.44, 0.63)	1.93% (1.92, 1.94)	1.87% (1.70, 2.06)	5.84% (5.81, 5.87)
	Hand	48 174	0.01% (0.01, 0.03)	0.23% (0.23, 0.24)	0.06% (0.04, 0.08)	1.09% (1.08, 1.09)	0.25% (0.21, 0.30)	4.12% (4.10, 4.14)
	Pelvis	1708	0.88% (0.53, 1.44)	1.27% (1.26, 1.27)	2.52% (1.87, 3.37)	3.60% (3.58, 3.62)	5.39% (4.41, 6.56)	7.99% (7.95, 8.02)
	Femur	59 590	6.06% (5.87, 6.26)	2.70% (2.69, 2.71)	12.65% (12.38, 12.92)	5.67% (5.65, 5.69)	23.69% (23.35, 24.03)	10.86% (10.82, 10.90)
	Tibia	32 253	0.29% (0.24, 0.36)	1.37% (1.36, 1.37)	0.69% (0.61, 0.79)	3.26% (3.24, 3.28)	1.82% (1.68, 1.97)	7.65% (7.61, 7.68)
	Fibula	21 450	0.11% (0.07, 0.16)	0.67% (0.67, 0.68)	0.23% (0.18, 0.31)	1.77% (1.75, 1.78)	0.69% (0.59, 0.81)	4.84% (4.81, 4.86)
	Tarsus	4645	0.04% (0.01, 0.16)	0.25% (0.25, 0.25)	0.09% (0.03, 0.22)	1.02% (1.01, 1.03)	0.47% (0.31, 0.72)	4.73% (4.70, 4.76)
	Foot	5521	0.11% (0.05, 0.24)	0.90% (0.90, 0.91)	0.16% (0.09, 0.31)	1.61% (1.60, 1.62)	0.60% (0.43, 0.84)	5.32% (5.29, 5.34)
Secondary open reduction	Overall	55 322	0.26% (0.22, 0.31)	2.10%(2.09, 2.11)	0.59% (0.53, 0.66)	4.51% (4.48, 4.53)	1.63% (1.53, 1.74)	9.32% (9.28, 9.35)
	Shoulder girdle	1343	0.00% (0.00, 0.29)	0.22% (0.21, 0.22)	0.07% (0.01, 0.42)	1.11% (1.11, 1.12)	0.52% (0.25, 1.07)	4.52% (4.50, 4.54)
	Humerus	3746	0.21% (0.11, 0.42)	1.60% (1.59, 1.61)	0.80% (0.56, 1.14)	4.10% (4.08, 4.12)	2.72% (2.25, 3.29)	8.85% (8.81, 8.89)
	Radius	29 718	0.04% (0.03, 0.07)	0.32% (0.32, 0.32)	0.16% (0.12, 0.21)	1.08% (1.08, 1.09)	0.84% (0.74, 0.95)	4.26% (4.23, 4.28)
	Ulna	1597	0.25% (0.10, 0.64)	0.59% (0.59, 0.59)	0.44% (0.21, 0.90)	1.64% (1.63, 1.65)	0.94% (0.57, 1.54)	5.41% (5.37, 5.43)
	Hand	4788	0.00% (0.00, 0.08)	0.20% (0.20, 0.20)	0.00% (0.00, 0.08)	0.92% (0.91, 0.92)	0.13% (0.06, 0.27)	3.79% (3.77, 3.82)
	Pelvis	139	0.00% (0.00, 2.69)	1.08% (1.07, 1.09)	0.00% (0.00, 2.69)	3.10% (3.08, 3.11)	1.44% (0.40, 5.09)	7.43% (7.39, 7.46)
	Femur	3249	2.80% (2.29, 3.43)	2.33% (2.32, 2.34)	5.82% (5.06, 6.68)	4.93% (4.91, 4.95)	11.60% (10.55, 12.75)	10.15% (10.11, 10.19)
	Tibia	4104	0.46% (0.30, 0.72)	1.17% (1.16, 1.17)	0.88% (0.63, 1.21)	2.80% (2.78, 2.82)	1.97% (1.59, 2.45)	7.11% (7.07, 7.13)
	Fibula	4963	0.14% (0.07, 0.29)	0.57% (0.57, 0.58)	0.28% (0.17, 0.47)	1.50% (1.49, 1.51)	0.77% (0.56, 1.05)	4.47% (4.44, 4.49)
	Tarsus	458	0.00% (0.00, 0.83)	0.21% (0.21, 0.21)	0.00% (0.00, 0.83)	0.86% (0.86, 0.87)	1.53% (0.74, 3.12)	4.37% (4.35, 4.39)
	Foot	338	0.00% (0.00, 1.12)	0.77% (0.76, 0.77)	0.00% (0.00, 1.12)	1.37% (1.36, 1.38)	0.00% (0.00, 1.12)	4.91% (4.89, 4.94)
Closed reduction	Overall	222 047	4.88% (4.79, 4.97)	2.48% (2.46, 2.49)	10.48% (10.36, 10.61)	5.42% (5.39, 5.45)	19.60% (19.43, 19.76)	10.48% (10.44, 10.53)
	Shoulder girdle	325	0.00% (0.00, 1.17)	0.26% (0.26, 0.26)	0.62% (0.17, 2.22)	1.40% (1.39, 1.40)	0.92% (0.31, 2.68)	5.23% (5.20, 5.26)
	Humerus	2674	4.08% (3.39, 4.89)	1.91% (1.90, 1.92)	12.15% (10.97, 13.45)	4.98% (4.96, 5.00)	24.27% (22.68, 25.93)	10.04% (10.01, 10.08)
	Radius	16 808	0.11% (0.07, 0.17)	0.38% (0.38, 0.39)	0.29% (0.22, 0.39)	1.36% (1.35, 1.37)	1.30% (1.14, 1.49)	4.93% (4.90, 4.95)
	Ulna	595	0.17% (0.03, 0.95)	0.71% (0.71, 0.71)	0.50% (0.17, 1.47)	2.04% (2.03, 2.05)	1.85% (1.04, 3.28)	6.23% (6.20, 6.26)
	Hand	38 221	0.03% (0.02, 0.05)	0.24% (0.24, 0.24)	0.09% (0.06, 0.12)	1.15% (1.14, 1.16)	0.35% (0.30, 0.42)	4.40% (4.38, 4.43)
	Pelvis	315	0.95% (0.32, 2.76)	1.29% (1.29, 1.30)	1.90% (0.88, 4.09)	3.80% (3.78, 3.82)	5.71% (3.64, 8.85)	8.48% (8.45, 8.51)
	Femur	149 834	7.10% (6.97, 7.23)	2.76% (2.74, 2.77)	15.17% (14.99, 15.36)	5.95% (5.93, 5.98)	28.13% (27.91, 28.36)	11.47% (11.42, 11.51)
	Tibia	10 096	0.50% (0.38, 0.65)	1.39% (1.39, 1.40)	1.05% (0.87, 1.27)	3.44% (3.43, 3.46)	2.64% (2.35, 2.98)	8.12% (8.09, 8.16)
	Fibula	565	0.18% (0.03, 1.00)	0.69% (0.68, 0.69)	1.24% (0.60, 2.54)	1.87% (1.86, 1.88)	2.83% (1.75, 4.55)	5.17% (5.14, 5.19)
	Tarsus	654	0.00% (0.00, 0.58)	0.26% (0.25, 0.26)	0.31% (0.08, 1.11)	1.08% (1.08, 1.09)	0.61% (0.24, 1.56)	5.06% (5.03, 5.08)
	Foot	1524	0.20% (0.07, 0.58)	0.92% (0.92, 0.93)	0.33% (0.14, 0.77)	1.71% (1.70, 1.72)	1.31% (0.85, 2.02)	5.67% (5.65, 5.70)
Other internal fixation	Overall	14 006	1.69% (1.49, 1.92)	1.94% (1.93, 1.95)	5.76% (5.39, 6.16)	5.07% (5.04, 5.10)	13.58% (13.02, 14.16)	10.44% (10.39, 10.47)
	Shoulder girdle	420	0.00% (0.00, 0.91)	0.20% (0.20, 0.20)	0.00% (0.00, 0.91)	1.28% (1.28, 1.29)	0.48% (0.13, 1.72)	5.20% (5.17, 5.22)
	Humerus	744	2.55% (1.64, 3.95)	1.47% (1.46, 1.48)	9.01% (7.15, 11.28)	4.64% (4.61, 4.66)	23.12% (20.23, 26.28)	9.99% (9.95, 10.03)
	Radius	333	0.30% (0.05, 1.68)	0.29% (0.29, 0.29)	1.50% (0.64, 3.47)	1.25% (1.24, 1.26)	2.40% (1.22, 4.67)	4.90% (4.87, 4.93)
	Ulna	285	0.00% (0.00, 1.33)	0.54% (0.54, 0.54)	0.70% (0.19, 2.52)	1.88% (1.87, 1.89)	1.75% (0.75, 4.04)	6.19% (6.16, 6.22)
	Hand	3144	0.00% (0.00, 0.12)	0.18% (0.18, 0.18)	0.22% (0.11, 0.46)	1.06% (1.05, 1.07)	0.51% (0.31, 0.83)	4.38% (4.35, 4.40)
	Pelvis	133	0.00% (0.00, 2.81)	0.99% (0.99, 1.00)	0.00% (0.00, 2.81)	3.52% (3.50, 3.54)	1.50% (0.41, 5.32)	8.43% (8.40, 8.47)
	Femur	5033	4.11% (3.60, 4.70)	2.15% (2.14, 2.16)	13.77% (12.84, 14.75)	5.56% (5.53, 5.58)	31.97% (30.69, 33.27)	11.41% (11.38, 11.45)
	Tibia	1280	0.47% (0.22, 1.02)	1.07% (1.06, 1.08)	1.95% (1.33, 2.87)	3.19% (3.18, 3.21)	4.92% (3.87, 6.25)	8.08% (8.04, 8.11)
	Fibula	234	0.00% (0.00, 1.62)	0.52% (0.52, 0.53)	0.00% (0.00, 1.62)	1.72% (1.71, 1.73)	1.28% (0.44, 3.70)	5.13% (5.10, 5.16)
	Tarsus	587	0.00% (0.00, 0.65)	0.19% (0.19, 0.19)	0.17% (0.03, 0.96)	1.00% (0.99, 1.00)	0.68% (0.27, 1.74)	5.03% (5.00, 5.06)
	Foot	1156	0.09% (0.02, 0.49)	0.70% (0.70, 0.71)	0.17% (0.05, 0.63)	1.58% (1.57, 1.59)	0.61% (0.29, 1.24)	5.64% (5.61, 5.66)

* Adjusted for age, sex, ethnic group, residence setting, comorbidities, hospital frailty risk score, type of admission, number of previous fixation procedures and year of surgery. Results for ‘Arm (other)’, ‘Leg (other)’ and ‘Other’ fracture regions are not shown for brevity. Full table can be found as online supplemental table I

**Table 5:** Restricted mean time lost

Procedure	Fracture region	Restricted mean time lost (days)		
		At 1 year	At 3 years	At 5 years
Primary open reduction	Overall	12.96 (12.96, 12.97)	71.33 (71.26, 71.39)	164.47 (164.29, 164.64)
	Shoulder girdle	0.70 (0.70, 0.70)	7.10 (7.09, 7.11)	20.38 (20.35, 20.40)
	Humerus	10.96 (10.94, 10.98)	66.41 (66.24, 66.59)	161.35 (160.81, 161.89)
	Radius	1.39 (1.39, 1.40)	14.03 (14.02, 14.04)	43.09 (43.03, 43.14)
	Ulna	3.57 (3.56, 3.57)	32.50 (32.42, 32.58)	92.06 (91.76, 92.37)
	Hand	0.46 (0.46, 0.46)	4.97 (4.97, 4.97)	14.65 (14.63, 14.66)
	Arm (other)	2.74 (2.71, 2.77)	18.22 (17.96, 18.47)	53.80 (52.76, 54.84)
	Pelvis	13.35(13.22, 13.47)	74.17 (73.22, 75.11)	175.98 (173.17, 178.78)
	Femur	59.72 (59.51, 59.93)	304.35 (302.84, 305.87)	669.60 (665.31, 673.90)
	Tibia	3.93 (3.93, 3.93)	25.70 (25.66, 25.75)	66.35 (66.21, 66.50)
	Fibula	1.42 (1.41, 1.42)	10.69 (10.67, 10.70)	30.17 (30.11, 30.22)
	Leg (other)	2.56 (2.56, 2.56)	23.38 (23.32, 23.44)	66.95 (66.73, 67.17)
	Tarsus	0.91 (0.91, 0.91)	8.45 (8.43, 8.47)	26.48 (26.38, 26.59)
	Foot	1.02 (1.02, 1.02)	9.89 (9.87, 9.92)	27.18 (27.09, 27.27)
	Other	5.59 (5.53, 5.66)	33.56 (33.00, 34.13)	78.48 (76.86, 80.10)
Secondary open reduction	Overall	3.46 (3.46, 3.46)	25.68 (25.64, 25.71)	69.67 (69.54, 69.79)
	Shoulder girdle	0.73 (0.73, 0.73)	8.30 (8.26, 8.34)	24.61 (24.44, 24.78)
	Humerus	5.40 (5.38, 5.42)	42.53 (42.25, 42.82)	116.24 (115.18, 117.31)
	Radius	1.47 (1.47, 1.47)	15.40 (15.38, 15.43)	46.33 (46.24, 46.43)
	Ulna	1.97 (1.96, 1.98)	22.27 (22.09, 22.44)	69.90 (69.11, 70.68)
	Hand	0.09 (0.09, 0.09)	2.97 (2.96, 2.97)	11.12 (11.09, 11.15)
	Arm (other)	0.81 (0.78, 0.83)	14.45 (13.96, 14.93)	69.23 (64.56, 73.89)
	Pelvis	3.81 (3.73, 3.89)	40.62 (39.16, 42.08)	90.00 (86.35, 93.65)
	Femur	27.96 (27.68, 28.23)	156.85 (154.65, 159.05)	373.25 (366.43, 380.08)
	Tibia	4.64 (4.62, 4.66)	28.44 (28.30, 28.58)	73.22 (72.74, 73.70)
	Fibula	1.57 (1.56, 1.57)	13.67 (13.63, 13.72)	40.47 (40.29, 40.66)
	Leg (other)	4.66 (4.62, 4.70)	33.31 (32.89, 33.73)	92.21 (90.63, 93.80)
	Tarsus	1.53 (1.52, 1.54)	24.12 (23.75, 24.50)	59.61 (58.53, 60.69)
	Foot	0.00 (0.00, 0.00)	6.46 (6.39, 6.54)	21.57 (21.26, 21.88)
	Other	9.33 (8.58, 10.08)	38.55 (35.47, 41.63)	67.77(62.35, 73.19)
Closed reduction	Overall	49.29 (49.21, 49.37)	250.35 (249.78, 250.91)	545.50 (543.98, 547.02)
	Shoulder girdle	2.05 (2.03, 2.06)	11.92 (11.78, 12.06)	26.93 (26.57, 27.30)
	Humerus	59.39 (58.38, 60.40)	276.51 (270.43, 282.59)	559.12 (545.03, 573.22)
	Radius	2.37 (2.36, 2.37)	22.02 (21.97, 22.07)	67.12 (66.91, 67.34)
	Ulna	3.35 (3.32, 3.38)	29.67 (29.26, 30.09)	100.49 (98.27, 102.72)
	Hand	0.67 (0.67, 0.67)	6.03 (6.02, 6.03)	18.27 (18.25, 18.29)
	Arm (other)	3.43 (3.37, 3.50)	16.08 (15.70, 16.45)	40.45 (39.24, 41.66)
	Pelvis	10.50 (10.30, 10.70)	88.99 (85.92, 92.07)	202.57 (194.23, 210.92)
	Femur	71.00 (70.82, 71.17)	359.20 (357.92, 360.48)	782.71 (779.06, 786.36)
	Tibia	6.03 (6.02, 6.05)	36.22 (36.09, 36.35)	88.33 (87.93, 88.73)
	Fibula	6.75 (6.67, 6.83)	39.44 (38.81, 40.07)	91.43 (89.68, 93.18)
	Leg (other)	11.54 (11.28, 11.79)	61.98 (60.20, 63.77)	151.12 (145.69, 156.55)
	Tarsus	1.46 (1.45, 1.47)	12.16 (12.06, 12.27)	33.18 (32.81, 33.56)
	Foot	2.28 (2.27, 2.29)	17.55 (17.44, 17.67)	41.52 (41.19, 41.84)
	Other	14.25 (13.47, 15.03)	86.02 (79.25, 92.79)	227.34 (202.00, 252.67)
Other internal fixation	Overall	31.50 (31.33, 31.66)	163.88 (162.74, 165.02)	340.44 (337.73, 343.15)
	Shoulder girdle	0.14 (0.14, 0.14)	8.84 (8.74, 8.93)	28.61 (28.22, 29.01)
	Humerus	52.26 (50.67, 53.84)	294.62 (281.55, 307.70)	630.91 (596.82, 665.00)
	Radius	6.34 (6.25, 6.43)	36.91 (36.17, 37.65)	93.48 (91.06, 95.91)
	Ulna	3.36 (3.32, 3.40)	23.51 (23.09, 23.93)	53.96 (52.83, 55.10)
	Hand	1.15 (1.15, 1.15)	9.81 (9.78, 9.85)	29.03 (28.89, 29.17)
	Arm (other)	0.00 (0.00, 0.00)	0.00 (0.00, 0.00)	26.73 (25.76, 27.70)
	Pelvis	3.58 (3.51, 3.65)	39.61 (38.18, 41.05)	106.93 (101.95, 111.91)
	Femur	74.55 (73.46, 75.63)	376.47 (368.80, 384.14)	762.89 (744.56, 781.23)
	Tibia	11.46 (11.34, 11.57)	64.90 (64.01, 65.80)	147.25 (144.83, 149.66)
	Fibula	2.12 (2.10, 2.15)	18.59 (18.26, 18.92)	50.10 (48.98, 51.21)
	Leg (other)	5.13 (5.06, 5.19)	32.14 (31.52, 32.76)	80.00 (78.03, 81.97)
	Tarsus	0.83 (0.83, 0.84)	11.99 (11.88, 12.11)	42.17 (41.57, 42.77)
	Foot	1.24 (1.24, 1.24)	10.48 (10.41, 10.54)	28.82 (28.60, 29.04)
	Other	5.11 (5.03, 5.19)	38.43 (37.42, 39.44)	119.86 (115.12, 124.59)

Case-mix-adjusted pORIF overall mortality rates were 2% within 30 days, 5% within 90 days and 10% within 365 days; for CRIF, 2% within 30 days, 5% within 90 days and 10% within 365 days; and for sORIF, 2% within 30 days, 5% within 90 days and 9% within 365 days (tables 3 and 4).

### Sensitivity analysis

In sensitivity analyses excluding patients with neoplastic processes, the overall pattern of mortality following internal fixation was largely preserved, with femur fractures and closed reduction remaining the fracture region and procedure, respectively, with the highest observed crude mortality. However, mortality rates were attenuated across several categories, most notably for humerus and femur fractures (online supplemental tables G and H). For humerus fractures, crude 1-year mortality decreased from 6% to 3% and adjusted 1-year mortality from 10% to 7%; for femur fractures, crude 1-year mortality decreased from 27% to 24% and adjusted 1-year mortality from 11% to 9%. By procedure type, the largest attenuation was observed for ‘other internal fixation’, with crude 1-year mortality decreasing from 14% to 2% and adjusted 1-year mortality decreasing from 10% to 8%. However, less than 2% of fractures treated using this procedure were recorded as malignancy-associated pathological fractures. The marked attenuation, therefore, cannot be attributed solely to explicitly coded malignancy-associated pathological fractures. Because the sensitivity analysis excluded all patients with evidence of a neoplastic process, the change may reflect the broader mortality burden associated with underlying neoplastic disease, including among patients whose fracture was not recorded as pathological. Despite these changes, the mortality patterns identified in the primary analysis were largely preserved.

## Discussion

### Summary of key results and interpretation

In this study, all-cause mortality following internal fixation after different modalities of orthopaedic operative reduction of bone fractures was investigated in the context of the UK healthcare system. While the overall crude mortality rate within the first postoperative year was 1 in 10 patients, there was a wide variation by fracture region and procedure. For example, the overall crude mortality at 1 year following pORIF was 5%, with lower observed mortality for hand fractures (0.3%) and higher observed mortality for those occurring in classic osteoporotic sites, such as radius (1%), tibia (2%), humerus (5%) and femur (24%). Likewise, the overall RMTL was 164 days at 5 years, but varied from 15 days for hand fractures, 43 days for radial fractures, 66 days for tibial fractures, 161 days for humeral fractures and 670 days for femoral fractures. Similar patterns were observed following sORIF, CRIF and other internal fixation procedures.

The mortality rates reported in this study should be interpreted as all-cause mortality following internal fixation, not as mortality directly attributable to the fracture or the fixation procedure. This distinction is particularly important for patients with malignancy, in whom a fracture may occur as part of a broader oncological disease process, and subsequent death may be primarily related to the underlying neoplastic process rather than to skeletal trauma or surgery.

### Interpretation of adjusted estimates

Disparities in mortality rates across fracture regions and procedures were smaller in case-mix-adjusted estimates. However, the direction and magnitude of case-mix-adjusted estimates differed between fracture regions and procedures. For example, the adjusted overall 1-year mortality for humeral fractures (10%) exceeded the observed mortality (6%), whereas for femoral fractures, the adjusted mortality (11%) was lower than the observed (27%). Similarly, the adjusted overall 1-year mortality for pORIF (10%) exceeded the observed mortality (5%), whereas the adjusted estimate for CRIF (10%) was lower than the observed mortality (20%). These case-mix-adjusted estimates represent mortality risk under a common population structure and covariate distribution and require careful interpretation. Adjustment may decrease estimated mortality in groups with an adverse observed case mix (eg, older, frailer, more comorbid) and increase it in groups with a more favourable observed case mix. Therefore, adjusted estimates are presented to enable a more comparable assessment of mortality rates across groups while accounting for measured differences in baseline patient characteristics. However, adjusted mortality rates should not be interpreted as proof of an inherently higher mortality because residual confounding from fracture complexity, physiological status and other unmeasured factors remains possible.

### Interpretation of femoral-fracture mortality

In routine orthopaedic practice, hip and proximal femoral fracture patients represent a distinct, high-risk population characterised by older age, frailty, multimorbidity, immobility and vulnerability to medical complications. Therefore, the high observed mortality following femoral fracture fixation should not be interpreted simply as mortality caused by fixation. Rather, femoral fracture fixation often occurs in patients with substantial baseline physiological vulnerability, for whom the fracture is a sentinel event in a broader trajectory of declining health. This interpretation is supported by the attenuated differences in the case-mix-adjusted estimates and by the concentration of observed deaths among femoral fracture patients.

### Interpretation of sensitivity analysis

Admissions with pathological fractures were intentionally included in the primary analysis because fracture aetiology was not part of the eligibility criteria and the study aimed to provide nationally representative descriptive estimates for admissions involving an isolated fracture treated with internal fixation. Nevertheless, pathological fractures represented less than 2% of all admissions, and malignancy-associated pathological fractures accounted for only 0.7%. The sensitivity analysis excluded the broader population of patients with any recorded neoplastic process and should, therefore, not be interpreted as a direct comparison of pathological and traumatic fractures. This distinction is particularly relevant to the ‘other internal fixation’ category. Although crude 1-year mortality in this group decreased from 14% to 2% following the exclusion of patients with neoplastic processes, less than 2% of admissions in this procedure group were recorded as malignancy-associated pathological fractures. The attenuation may, therefore, reflect the wider mortality burden of an underlying oncological process rather than an unusually high prevalence of pathological fractures. The principal mortality patterns were otherwise preserved, supporting the robustness of the main descriptive findings.

Given the low prevalence of malignancy-associated pathological fractures and the scope of the present study, separate analyses investigating pathological fractures were not undertaken. Such comparisons would require a dedicated analysis and should be addressed in future research.

### Comparison with current literature

Except for proximal femoral fractures, there is scarce information on all-cause mortality following fracture internal fixation.

Mortality after humeral fractures treated with internal fixation has been reported to range between 0.4% and 2.1% within the first 30 days^59–62^ and between 1.5% and 3.5%^62 63^ within 90 days postprocedure, which is consistent with our findings of 1% at 30 days and 3% at 90 days. Information on 1-year mortality is less clear. Duey *et al*^62^ conducted a retrospective study using US Medicare data to compare open reduction internal fixation (ORIF) for proximal humeral fractures with total shoulder arthroplasty, hemiarthroplasty and non-operative treatment. They reported a 4% mortality rate after 1 year, which is lower than in our cohort (6%), despite having an older population. Conversely, a German study^52^ and a Finnish study^64^ reported a 1-year mortality after humeral fractures of 9%, but only approximately 40% of the patients in their sample underwent ORIF, making it hard to compare with our population.

Only three studies were identified reporting mortality after internal fixation for radial fractures.^65–67^ These studies reported a 30-day mortality rate ranging from 0.06% to 0.2%, which is consistent with our observed mortality rate of 0.07%. However, only the study by Schick *et al*^65^ had a primary aim of describing overall mortality after internal fixation of radial fractures. Shauver *et al*^68^ reported a 3% overall mortality after distal radial fractures, which is three times higher than the 0.9% reported in our population. This study used data from 2007 and included surgical and conservative management approaches.

Mortality after internal fixation for tibial fractures has been reported at 30 days postprocedure to be between 0.2% and 1.6%,^69–72^ which is consistent with our findings of a 30-day mortality rate of 0.4%, with the higher mortality of 1.6% reported by Pollard *et al*^72^ likely due to restricting the analysis to patients between 65 and 89 years old. Only one Canadian study reported mortality beyond 30 days,^73^ which reported a 90-day mortality rate of 0.9%. This is similar to the 0.8% mortality rate within the same period identified in this study.

### Strengths and limitations

A major strength of this study is its use of a large nationwide dataset providing virtually complete coverage of NHS hospital admissions and procedures in England. In addition, mandatory death registration enabled near-complete ascertainment of mortality. However, this study also has several limitations. First, diagnoses, comorbidities, fracture regions and procedures were derived from ICD-10 and OPCS-4 codes recorded for administrative purposes. Coding errors, differences in coding practice between centres and limited coding granularity may therefore have resulted in misclassification. Furthermore, procedures were not independently clinically adjudicated and OPCS-4 codes may not fully capture procedural intent or the sequence of previous fracture treatments. For example, the ‘secondary open reduction’ category should not be interpreted as synonymous with revision fixation in the clinical sense.

Second, although key factors that have been previously associated with complications and mortality after orthopaedic procedures were included, information on other clinically important factors, such as socioeconomic status,^49^ medications,^49 74^ fracture pattern,^75^ wound status,^76^ preoperative American Society of Anaesthesiology Classification^69 77^ or intraoperative details,^78–80^ was not available for analysis. Data on implant type, surgeon volume and hospital-level characteristics were also unavailable. Their absence may result in residual confounding since these characteristics influence both the selected fixation procedure and subsequent mortality. However, the direction of this residual confounding cannot be reliably determined. For instance, if patients with higher physiological instability or more complex fractures were preferentially treated with a particular procedure, the adjusted mortality estimates for this procedure could be biased upwards. Conversely, procedures with a higher concentration of patients with more favourable conditions could bias estimates downwards. These limitations need to be considered when comparing adjusted estimates across fracture regions and procedures.

Third, when estimating adjusted mortality rates, non-linear relationships for quantitative variables or potential clinically meaningful interactions, such as those between procedure and the anatomical region of the fracture, were not considered.

The aforementioned limitations primarily affect the interpretation of case-mix-adjusted comparisons rather than the observed all-cause mortality, which was based on virtually complete ascertainment. Therefore, the adjusted mortality estimates should be regarded as population-level standardised descriptions conditional on the measured covariates, rather than estimates that fully account for clinical severity and procedural complexity.

Finally, although the RMTL provides a clinically interpretable measure of mortality within a specified horizon, because the study did not have a comparable general-population cohort or a non-operative fracture cohort, the RMTL estimates should be interpreted solely as descriptive summaries of mortality within each fracture and procedure group rather than excess time lost relative to an external reference population.

## Conclusions

This national retrospective cohort study provides descriptive crude and case-mix-adjusted estimates of all-cause mortality rates following internal fixation for isolated non-polytrauma fractures. Mortality varied substantially across fracture regions, with femoral fractures accounting for most deaths, although case-mix-adjusted estimates showed less variation than crude rates, suggesting that mortality rates should be interpreted in the context of patient characteristics such as age, frailty and comorbidities rather than as mortality directly caused by fracture fixation.

These findings help to fill the existing knowledge gap in outcomes after internal fixation of fractures and provide population-level context for mortality following internal fixation. The reported mortality rates should help inform clinical decisions and assist healthcare professionals during the process of obtaining informed consent as well as help manage patients’ expectations. However, the administrative nature of the data fails to capture important determinants of individual prognosis, such as physiological status, detailed fracture morphology or implant characteristics. Therefore, these estimates should not be used in isolation.

The mortality rates and RMTL estimates reported in this study should inform the planning of future clinical research involving patients undergoing fracture fixation. Information on mortality at different horizons provides estimates of expected event frequencies that can support feasibility assessments, sample-size calculations, selection of clinically appropriate endpoints and follow-up periods and anticipation of attrition due to death.

These estimates are descriptive and should not be interpreted as causal treatment-effect estimates for internal fixation, as causal comparisons between procedures, as survival losses attributable specifically to internal fixation, or as individualised prognostic estimates.

## Supplementary material

10.1136/bmjopen-2026-122170 online supplemental file 1

10.1136/bmjopen-2026-122170 online supplemental file 2

10.1136/bmjopen-2026-122170 online supplemental file 3

10.1136/bmjopen-2026-122170 online supplemental file 4

10.1136/bmjopen-2026-122170 online supplemental file 5

10.1136/bmjopen-2026-122170 online supplemental file 6

10.1136/bmjopen-2026-122170 online supplemental file 7

10.1136/bmjopen-2026-122170 online supplemental file 8

10.1136/bmjopen-2026-122170 online supplemental file 9

## Data Availability

Data may be obtained from a third party and are not publicly available.
