# Supplementary material for: All-cause mortality following internal fixation for isolated fractures in non-polytrauma admissions: a comprehensive 10-year nationwide linked data retrospective cohort study
Source: BMJ Open. 2026 Sep 8;16(9):e122170. doi: 10.1136/bmjopen-2026-122170 (PMC13560970; doi:10.1136/bmjopen-2026-122170)
Supplement: Supplementary file 1 [file bmjopen-16-9-s009.pdf]

Table 1.- Crude and adjusted post-operative mortality rates by procedure

**Procedure**	**Fracture region**	**Admissions**	**Within 30 days**		**Within 90 days**		**Within 365 days**	
			**Crude**	**Adjusted***	**Crude**	**Adjusted***	**Crude**	**Adjusted***
Primary open reduction	Overall	339,383	1.22% [1.18, 1.26]	2.43% [2.42, 2.45]	2.64% [2.59, 2.70]	5.17% [5.14, 5.19]	5.34% [5.27, 5.42]	9.94% [9.90, 9.99]
	Shoulder girdle	29,465	0.02% [0.01, 0.05]	0.25% [0.25, 0.26]	0.10% [0.07, 0.14]	1.32% [1.31, 1.32]	0.41% [0.34, 0.49]	4.90% [4.87, 4.92]
	Humerus	36,041	0.72% [0.64, 0.82]	1.87% [1.86, 1.88]	2.05% [1.91, 2.20]	4.74% [4.71, 4.76]	4.88% [4.66, 5.10]	9.48% [9.45, 9.52]
	Radius	61,831	0.07% [0.05, 0.09]	0.38% [0.37, 0.38]	0.21% [0.18, 0.25]	1.28% [1.27, 1.29]	0.78% [0.71, 0.85]	4.61% [4.59, 4.64]
	Ulna	22,099	0.20% [0.15, 0.27]	0.70% [0.69, 0.70]	0.52% [0.44, 0.63]	1.93% [1.92, 1.94]	1.87% [1.70, 2.06]	5.84% [5.81, 5.87]
	Hand	48,174	0.01% [0.01, 0.03]	0.23% [0.23, 0.24]	0.06% [0.04, 0.08]	1.09% [1.08, 1.09]	0.25% [0.21, 0.30]	4.12% [4.10, 4.14]
	Arm (other)	347	0.00% [0.00, 1.09]	0.89% [0.89, 0.90]	0.29% [0.05, 1.61]	1.87% [1.86, 1.88]	1.73% [0.79, 3.72]	6.10% [6.07, 6.12]
	Pelvis	1,708	0.88% [0.53, 1.44]	1.27% [1.26, 1.27]	2.52% [1.87, 3.37]	3.60% [3.58, 3.62]	5.39% [4.41, 6.56]	7.99% [7.95, 8.02]
	Femur	59,590	6.06% [5.87, 6.26]	2.70% [2.69, 2.71]	12.65% [12.38, 12.92]	5.67% [5.65, 5.69]	23.69% [23.35, 24.03]	10.86% [10.82, 10.90]
	Tibia	32,253	0.29% [0.24, 0.36]	1.37% [1.36, 1.37]	0.69% [0.61, 0.79]	3.26% [3.24, 3.28]	1.82% [1.68, 1.97]	7.65% [7.61, 7.68]
	Fibula	21,450	0.11% [0.07, 0.16]	0.67% [0.67, 0.68]	0.23% [0.18, 0.31]	1.77% [1.75, 1.78]	0.69% [0.59, 0.81]	4.84% [4.81, 4.86]
	Leg (other)	15,822	0.17% [0.12, 0.25]	0.64% [0.64, 0.64]	0.42% [0.33, 0.53]	1.76% [1.74, 1.77]	1.35% [1.18, 1.54]	5.32% [5.29, 5.35]
	Tarsus	4,645	0.04% [0.01, 0.16]	0.25% [0.25, 0.25]	0.09% [0.03, 0.22]	1.02% [1.01, 1.03]	0.47% [0.31, 0.72]	4.73% [4.70, 4.76]
	Foot	5,521	0.11% [0.05, 0.24]	0.90% [0.90, 0.91]	0.16% [0.09, 0.31]	1.61% [1.60, 1.62]	0.60% [0.43, 0.84]	5.32% [5.29, 5.34]
	Other	437	0.23% [0.04, 1.28]	1.32% [1.31, 1.33]	1.14% [0.49, 2.65]	3.32% [3.30, 3.33]	2.52% [1.41, 4.45]	6.59% [6.56, 6.62]
Secondary open reduction	Overall	55,322	0.26% [0.22, 0.31]	2.10% [2.09, 2.11]	0.59% [0.53, 0.66]	4.51% [4.48, 4.53]	1.63% [1.53, 1.74]	9.32% [9.28, 9.35]
	Shoulder girdle	1,343	0.00% [0.00, 0.29]	0.22% [0.21, 0.22]	0.07% [0.01, 0.42]	1.11% [1.11, 1.12]	0.52% [0.25, 1.07]	4.52% [4.50, 4.54]
	Humerus	3,746	0.21% [0.11, 0.42]	1.60% [1.59, 1.61]	0.80% [0.56, 1.14]	4.10% [4.08, 4.12]	2.72% [2.25, 3.29]	8.85% [8.81, 8.89]
	Radius	29,718	0.04% [0.03, 0.07]	0.32% [0.32, 0.32]	0.16% [0.12, 0.21]	1.08% [1.08, 1.09]	0.84% [0.74, 0.95]	4.26% [4.23, 4.28]
	Ulna	1,597	0.25% [0.10, 0.64]	0.59% [0.59, 0.59]	0.44% [0.21, 0.90]	1.64% [1.63, 1.65]	0.94% [0.57, 1.54]	5.41% [5.37, 5.43]
	Hand	4,788	0.00% [0.00, 0.08]	0.20% [0.20, 0.20]	0.00% [0.00, 0.08]	0.92% [0.91, 0.92]	0.13% [0.06, 0.27]	3.79% [3.77, 3.82]
	Arm (other)	61	0.00% [0.00, 5.92]	0.76% [0.76, 0.77]	0.00% [0.00, 5.92]	1.59% [1.58, 1.60]	1.64% [0.29, 8.72]	5.64% [5.61, 5.67]
	Pelvis	139	0.00% [0.00, 2.69]	1.08% [1.07, 1.09]	0.00% [0.00, 2.69]	3.10% [3.08, 3.11]	1.44% [0.40, 5.09]	7.43% [7.39, 7.46]
	Femur	3,249	2.80% [2.29, 3.43]	2.33% [2.32, 2.34]	5.82% [5.06, 6.68]	4.93% [4.91, 4.95]	11.60% [10.55, 12.75]	10.15% [10.11, 10.19]
	Tibia	4,104	0.46% [0.30, 0.72]	1.17% [1.16, 1.17]	0.88% [0.63, 1.21]	2.80% [2.78, 2.82]	1.97% [1.59, 2.45]	7.11% [7.07, 7.13]
	Fibula	4,963	0.14% [0.07, 0.29]	0.57% [0.57, 0.58]	0.28% [0.17, 0.47]	1.50% [1.49, 1.51]	0.77% [0.56, 1.05]	4.47% [4.44, 4.49]

	Leg (other)	793	0.25% [0.07, 0.91]	0.54% [0.54, 0.55]	0.63% [0.27, 1.47]	1.49% [1.48, 1.50]	2.27% [1.44, 3.56]	4.92% [4.89, 4.95]
	Tarsus	458	0.00% [0.00, 0.83]	0.21% [0.21, 0.21]	0.00% [0.00, 0.83]	0.86% [0.86, 0.87]	1.53% [0.74, 3.12]	4.37% [4.35, 4.39]
	Foot	338	0.00% [0.00, 1.12]	0.77% [0.76, 0.77]	0.00% [0.00, 1.12]	1.37% [1.36, 1.38]	0.00% [0.00, 1.12]	4.91% [4.89, 4.94]
	Other	25	0.00% [0.00, 13.32]	1.13% [1.12, 1.14]	0.00% [0.00, 13.32]	2.85% [2.84, 2.87]	4.00% [0.71, 19.54]	6.11% [6.08, 6.14]
Closed reduction	Overall	222,047	4.88% [4.79, 4.97]	2.48% [2.46, 2.49]	10.48% [10.36, 10.61]	5.42% [5.39, 5.45]	19.60% [19.43, 19.76]	10.48% [10.44, 10.53]
	Shoulder girdle	325	0.00% [0.00, 1.17]	0.26% [0.26, 0.26]	0.62% [0.17, 2.22]	1.40% [1.39, 1.40]	0.92% [0.31, 2.68]	5.23% [5.20, 5.26]
	Humerus	2,674	4.08% [3.39, 4.89]	1.91% [1.90, 1.92]	12.15% [10.97, 13.45]	4.98% [4.96, 5.00]	24.27% [22.68, 25.93]	10.04% [10.01, 10.08]
	Radius	16,808	0.11% [0.07, 0.17]	0.38% [0.38, 0.39]	0.29% [0.22, 0.39]	1.36% [1.35, 1.37]	1.30% [1.14, 1.49]	4.93% [4.90, 4.95]
	Ulna	595	0.17% [0.03, 0.95]	0.71% [0.71, 0.71]	0.50% [0.17, 1.47]	2.04% [2.03, 2.05]	1.85% [1.04, 3.28]	6.23% [6.20, 6.26]
	Hand	38,221	0.03% [0.02, 0.05]	0.24% [0.24, 0.24]	0.09% [0.06, 0.12]	1.15% [1.14, 1.16]	0.35% [0.30, 0.42]	4.40% [4.38, 4.43]
	Arm (other)	102	0.98% [0.17, 5.35]	0.91% [0.91, 0.92]	0.98% [0.17, 5.35]	1.98% [1.97, 1.99]	0.98% [0.17, 5.35]	6.49% [6.46, 6.52]
	Pelvis	315	0.95% [0.32, 2.76]	1.29% [1.29, 1.30]	1.90% [0.88, 4.09]	3.80% [3.78, 3.82]	5.71% [3.64, 8.85]	8.48% [8.45, 8.51]
	Femur	149,834	7.10% [6.97, 7.23]	2.76% [2.74, 2.77]	15.17% [14.99, 15.36]	5.95% [5.93, 5.98]	28.13% [27.91, 28.36]	11.47% [11.42, 11.51]
	Tibia	10,096	0.50% [0.38, 0.65]	1.39% [1.39, 1.40]	1.05% [0.87, 1.27]	3.44% [3.43, 3.46]	2.64% [2.35, 2.98]	8.12% [8.09, 8.16]
	Fibula	565	0.18% [0.03, 1.00]	0.69% [0.68, 0.69]	1.24% [0.60, 2.54]	1.87% [1.86, 1.88]	2.83% [1.75, 4.55]	5.17% [5.14, 5.19]
	Leg (other)	280	0.36% [0.06, 1.99]	0.65% [0.65, 0.66]	1.43% [0.56, 3.61]	1.86% [1.85, 1.87]	4.64% [2.73, 7.78]	5.68% [5.65, 5.70]
	Tarsus	654	0.00% [0.00, 0.58]	0.26% [0.25, 0.26]	0.31% [0.08, 1.11]	1.08% [1.08, 1.09]	0.61% [0.24, 1.56]	5.06% [5.03, 5.08]
	Foot	1,524	0.20% [0.07, 0.58]	0.92% [0.92, 0.93]	0.33% [0.14, 0.77]	1.71% [1.70, 1.72]	1.31% [0.85, 2.02]	5.67% [5.65, 5.70]
	Other	54	1.85% [0.33, 9.77]	1.35% [1.34, 1.36]	1.85% [0.33, 9.77]	3.50% [3.48, 3.52]	5.56% [1.91, 15.11]	7.01% [6.99, 7.04]
Other internal reduction	Overall	14,006	1.69% [1.49, 1.92]	1.94% [1.93, 1.95]	5.76% [5.39, 6.16]	5.07% [5.04, 5.10]	13.58% [13.02, 14.16]	10.44% [10.39, 10.47]
	Shoulder girdle	420	0.00% [0.00, 0.91]	0.20% [0.20, 0.20]	0.00% [0.00, 0.91]	1.28% [1.28, 1.29]	0.48% [0.13, 1.72]	5.20% [5.17, 5.22]
	Humerus	744	2.55% [1.64, 3.95]	1.47% [1.46, 1.48]	9.01% [7.15, 11.28]	4.64% [4.61, 4.66]	23.12% [20.23, 26.28]	9.99% [9.95, 10.03]
	Radius	333	0.30% [0.05, 1.68]	0.29% [0.29, 0.29]	1.50% [0.64, 3.47]	1.25% [1.24, 1.26]	2.40% [1.22, 4.67]	4.90% [4.87, 4.93]
	Ulna	285	0.00% [0.00, 1.33]	0.54% [0.54, 0.54]	0.70% [0.19, 2.52]	1.88% [1.87, 1.89]	1.75% [0.75, 4.04]	6.19% [6.16, 6.22]
	Hand	3,144	0.00% [0.00, 0.12]	0.18% [0.18, 0.18]	0.22% [0.11, 0.46]	1.06% [1.05, 1.07]	0.51% [0.31, 0.83]	4.38% [4.35, 4.40]
	Arm (other)	122	0.00% [0.00, 3.05]	0.70% [0.69, 0.70]	0.00% [0.00, 3.05]	1.83% [1.82, 1.84]	0.00% [0.00, 3.05]	6.46% [6.42, 6.48]
	Pelvis	133	0.00% [0.00, 2.81]	0.99% [0.99, 1.00]	0.00% [0.00, 2.81]	3.52% [3.50, 3.54]	1.50% [0.41, 5.32]	8.43% [8.40, 8.47]
	Femur	5,033	4.11% [3.60, 4.70]	2.15% [2.14, 2.16]	13.77% [12.84, 14.75]	5.56% [5.53, 5.58]	31.97% [30.69, 33.27]	11.41% [11.38, 11.45]
	Tibia	1,280	0.47% [0.22, 1.02]	1.07% [1.06, 1.08]	1.95% [1.33, 2.87]	3.19% [3.18, 3.21]	4.92% [3.87, 6.25]	8.08% [8.04, 8.11]

	Fibula	234	0.00% [0.00, 1.62]	0.52% [0.52, 0.53]	0.00% [0.00, 1.62]	1.72% [1.71, 1.73]	1.28% [0.44, 3.70]	5.13% [5.10, 5.16]
	Leg (other)	308	0.65% [0.18, 2.34]	0.50% [0.49, 0.50]	0.97% [0.33, 2.82]	1.72% [1.70, 1.73]	2.27% [1.11, 4.62]	5.65% [5.62, 5.67]
	Tarsus	587	0.00% [0.00, 0.65]	0.19% [0.19, 0.19]	0.17% [0.03, 0.96]	1.00% [0.99, 1.00]	0.68% [0.27, 1.74]	5.03% [5.00, 5.06]
	Foot	1,156	0.09% [0.02, 0.49]	0.70% [0.70, 0.71]	0.17% [0.05, 0.63]	1.58% [1.57, 1.59]	0.61% [0.29, 1.24]	5.64% [5.61, 5.66]
	Other	227	0.44% [0.08, 2.45]	1.04% [1.03, 1.04]	0.88% [0.24, 3.15]	3.25% [3.23, 3.27]	1.76% [0.69, 4.44]	6.97% [6.94, 7.01]

* Adjusted for age, sex, ethnic group, residence setting, comorbidities, hospital frailty risk score, type of admission, number of previous fixation procedures and year of surgery
