# Supplementary material for: All-cause mortality following internal fixation for isolated fractures in non-polytrauma admissions: a comprehensive 10-year nationwide linked data retrospective cohort study
Source: BMJ Open. 2026 Sep 8;16(9):e122170. doi: 10.1136/bmjopen-2026-122170 (PMC13560970; doi:10.1136/bmjopen-2026-122170)
Supplement: Supplementary file 2 [file bmjopen-2026-122170.reviewer_comments.pdf]

PEER REVIEW HISTORY

BMJ Open publishes all reviews undertaken for accepted manuscripts. Reviewers are asked to complete a checklist review form and are provided with free text boxes to elaborate on their assessment. These free text comments are reproduced below.

ARTICLE DETAILS

Title (Provisional)

All-cause mortality following internal fixation for isolated fractures in non-polytrauma admissions: A comprehensive 10-year nationwide linked-data retrospective cohort study.

Authors

Muruet, Walter; Hing, Caroline; Aylin, Paul; Bottle, Alex; Musbahi, Omar

---

VERSION 1 - REVIEW

**Reviewer**	**1**
**Name**	**Yeh, Kuang-Ting**
**Affiliation**	**Hualien Tzu Chi Hospital Buddhist Tzu Chi Medical Foundation, Department of Orthopedics**
**Date**	**07-Jun-2026**
**COI**	**none**

This nationwide population-based study analyzed 630,758 non-polytrauma internal fixation admissions in England (2013–2023), reporting all-cause mortality and RMTL stratified by fracture region and procedure type. Femoral fractures carried the highest mortality risk, though case-mix adjustment revealed patient-level factors—particularly age, frailty, and comorbidities—as the primary mortality drivers. I have some comments:

1. The manuscript occasionally employs language that implies causality, which is inconsistent with its observational, retrospective design. For instance, phrases such as "mortality associated with internal fixation" in the title and throughout the text may mislead readers into attributing deaths directly to the surgical procedure, when the study design only permits association. The authors should carefully revise all such language to reflect the descriptive and associative nature of the study, clearly distinguishing between mortality following internal fixation and mortality caused by it. This is particularly important given that surgical complications accounted for less than 0.1% of deaths, underscoring that the observed mortality is predominantly driven by the patient's underlying condition rather than the intervention itself.

2. While the authors appropriately perform case-mix adjustment, the interpretation of the adjusted results warrants more rigorous discussion. Notably, the adjusted 1-year mortality for humerus fractures (9.81%) exceeds its crude rate (6.20%), while the adjusted femur mortality (11.20%) is substantially lower than its crude rate (26.76%). This counter-intuitive finding—suggesting that humerus fractures may carry a higher relative risk than crude data imply—is briefly mentioned but not adequately explained. The authors should provide a more thorough mechanistic discussion of why adjustment reverses the relative risk ranking for certain fracture sites, and whether this reflects confounding by indication, differences in surgical complexity, or other clinical factors. Without this, readers may misinterpret the adjusted figures.

3. The RMTL is presented as a key outcome measure, yet its clinical applicability is not sufficiently contextualized for a general readership. The authors should clarify the assumptions underlying RMTL calculations, particularly regarding the choice of time horizons (1, 3, and 5 years) and how these were selected. Furthermore, the RMTL values for femoral fractures (669.60 days lost at 5 years) are strikingly high, yet no comparison is made to age-matched general population norms or to non-surgically managed fracture cohorts. Without such benchmarks, it is difficult for clinicians to translate these figures into meaningful patient counseling or informed consent discussions, which the authors identify as a primary application of their findings.

4. The sensitivity analysis excluding patients with neoplastic processes is a valuable addition, particularly given that malignancy accounts for 18% of all deaths. However, the analysis is incompletely reported and its implications are not fully explored. For example, the dramatic reduction in 1-year mortality for "Other Internal Fixation" from 13.58% to 2.18% after malignancy exclusion suggests that this subgroup is heavily confounded by pathological fractures, yet this is not explicitly discussed. The authors should expand the sensitivity analysis to include a broader discussion of pathological versus traumatic fractures, and ideally present stratified results for these two populations separately. Additionally, the authors should clarify whether patients with pathological fractures were intentionally included in the primary analysis and justify this decision.

5. The authors acknowledge several limitations related to administrative data, but the discussion of potential biases remains superficial and requires expansion. Specifically, the absence of ASA grade, specific fracture pattern (e.g., AO/OTA classification), implant type, surgeon volume, and hospital-level factors represents significant unmeasured confounding that could substantially affect the adjusted mortality estimates. Furthermore, the reliance on OPCS-4 and ICD-10 coding introduces the risk of misclassification bias, particularly for procedure type (e.g., distinguishing primary from revision fixation) and comorbidity ascertainment. The authors should quantify the extent of missing data where possible, discuss the direction and magnitude of likely biases, and explicitly state how these limitations affect the validity of their conclusions—especially regarding the generalizability of adjusted mortality estimates to individual clinical decision-making.

**Reviewer**	**2**
**Name**	**Brox, William**
**Affiliation**	**Timothy Brox, MD**
**Date**	**20-Jun-2026**
**COI**	**none**

The authors have investigated short- and long-term mortality following internal fixation in non-polytrauma patients with isolated fractures. They have completed and provided a STROBE reporting checklist. The methodology is described clearly and in appropriate detail, and the discussion of the analysis is thorough and well balanced.

The conclusions are appropriate and supported by the data analysis. This manuscript will be of interest to the general readership of the journal and represents a useful contribution to orthopaedic epidemiology.

VERSION 1 - AUTHOR RESPONSE

Reviewer 1 Comments

We thank the reviewer for their detailed and constructive feedback, which helped us greatly to improve the precision, interpretation and transparency of our manuscript.

Comment 1: Avoid causal language

The reviewer noted that some wording, such as "mortality associated with internal fixation," could imply that mortality was caused by the fixation procedure, which would be inappropriate for a retrospective observational study.

Response:

We agree and have conscientiously revised our manuscript to make a clear distinction between mortality following internal fixation and mortality caused by the fracture or orthopaedic procedure. The title, abstract, aims, interpretation and conclusions now consistently reiterate the non-causal nature of our study.

Specifically, we have made the following revisions to our manuscript (lines refer to the marked version of the main document):

- Revised the title (lines 1–3)
- Revised abstract (lines 51–73)
- Revised study description in the introduction (lines 131-134)
- Revised the discussion of the distinction between mortality following fixation and mortality attributable to fixation, and now it is presented earlier in the discussion section (lines 345–350)
- Expanded the discussion on the interpretation of adjusted estimates and residual confounding (lines 352–370)
- Added a caveat when discussing femoral fracture mortality (lines 374–376)
- Revised conclusions (lines 487- 493) and made explicit the non-causal nature of the study (lines 511–514)

Comment 2: Interpretation of the adjusted mortality estimates

The reviewer requested a more rigorous explanation of why adjustment increased mortality estimates for some groups, such as humeral fractures, while decreasing them substantially for others, such as femoral fractures.

Response:

We agree that the adjusted estimates required more careful explanation. We have therefore added a dedicated subsection within the discussion titled: **“**Interpretation of adjusted estimates.**”** In this section, we explain that the marginally standardised estimates represent mortality under a common population structure and measured covariate distribution. We also clarify that adjustment may reduce estimated mortality in groups with a relatively adverse case mix, while increasing estimates in groups with a comparatively favourable case mix. Furthermore, we also clarify that unmeasured factors may continue to confound the estimates.

We have also expanded the limitations section to explain the possible direction of residual confounding. For instance, preferential use of a procedure in patients with greater physiological instability or more complex fractures could bias the adjusted estimate upwards, whereas more favourable characteristics could bias it downwards. However, we acknowledge that the direction and magnitude of this bias cannot be reliably quantified using the available data.

Specifically, we have made the following revisions to our manuscript (lines refer to the marked version of the main document):

- New "Interpretation of adjusted estimates" subsection (lines 352–370)
- Expanded discussion of residual confounding and its potential direction (lines 457–466)
- Further clarification of the appropriate interpretation and validity of the adjusted estimates (lines 472–477)

<u>Comment 3: Expand the description of restricted mean time lost</u>

The reviewer requested further explanation of the restricted mean time lost, the selection of the one-, three- and five-year horizons, and the absence of comparison with age-matched general-population or non-operatively managed cohorts.

Response:

We agree and have expanded both the Methods and Discussion.

The revised Methods section explains how the restricted mean time lost (RMTL) is calculated and clarifies that the one-, three- and five-year horizons were selected a priori following clinical input to describe intermediate- and longer-term mortality burden. Five years was selected as the longest horizon because it was considered clinically meaningful while retaining adequate follow-up.

We agree that the femoral-fracture estimate cannot be interpreted adequately as excess survival loss without an appropriate reference population. However, the analysed dataset did not include a matched general-population cohort or a comparable non-operatively managed fracture cohort. Therefore, we considered it inappropriate to introduce an

indirect or potentially misleading external comparison. Instead, we have explicitly stated that the restricted mean time lost estimates are descriptive summaries within each fracture and procedure group and should not be interpreted as excess time lost relative to an external reference population. Comparison of RMTL with non-operatively managed fractures is an interesting question that deserves further research.

Furthermore, we have also clarified the proposed clinical application of these estimates. The conclusions now state that mortality and restricted mean time lost are meant to provide population-level context but should not be used in isolation. Their principal research applications include feasibility assessment, sample-size calculation, endpoint and follow-up selection, and anticipating attrition due to death.

Specifically, we have made the following revisions to our manuscript (lines refer to the marked version of the main document):

- Expanded definition and rationale for the restricted mean time lost horizons (lines 235–244)
- Discussed limitation concerning the absence of an external comparison cohort (lines 479–484)
- Clarified the applicability to the design of future clinical studies (lines 504–509)

<u>Comment 4: Sensitivity analysis and pathological fractures</u>

The reviewer requested more complete explanation of the sensitivity analysis, clarification of whether pathological fractures were intentionally included, and further consideration of the marked reduction in mortality in “Other Internal Fixation” after patients with neoplastic disease were excluded.

Response:

We agree and have substantially expanded the reporting and interpretation of this analysis. Pathological fractures were intentionally included in the primary analysis. Fracture aetiology was not used as an eligibility criterion because the study was designed to provide nationally representative descriptive estimates for all admissions involving an isolated fracture treated with internal fixation. We have now stated this explicitly in the

Methods section. Furthermore, we describe the prevalence of pathological fractures in the Results section. Pathological fractures accounted for fewer than 2% of the primary analysis sample, with malignancy-associated pathological fractures accounting for fewer than 1% of admissions.

Additionally, the revised sensitivity analysis section now reports the observed shifts in crude and adjusted mortality rates for the humerus, femur and "other internal fixation". We have addressed the reduction in crude one-year mortality for "other internal fixation". Furthermore, we clarify that because fewer than 2% of admissions undergoing "other internal fixation" were recorded as malignancy-associated pathological fractures, the attenuation therefore cannot be attributed solely to a high prevalence of recorded pathological fractures and likely reflects the broader mortality burden associated with underlying neoplastic processes.

Given the low prevalence of malignancy-associated pathological fractures and the primary scope of this study, we did not undertake separate stratified analyses. We agree that pathological fractures are clinically important, but a robust comparison would require a dedicated study with more detailed consideration that goes beyond the aims of this study. We have identified this as an area for future research.

Specifically, we have made the following revisions to our manuscript (lines refer to the marked version of the main document):

- Clarified that fractures were included regardless of aetiology (lines 184–186)
- Revised description of the sensitivity analysis (lines 255–256)
- Description of pathological fractures in the results (lines 269–272)
- Expanded reporting and interpretation of the sensitivity analysis (lines 309–328)
- Provided a dedicated interpretation of the sensitivity analyses (lines 382–403)

<u>Comment 5: Administrative-data limitations</u>

The reviewer requested an expanded discussion of unavailable clinical variables, coding-related misclassification, the likely direction and magnitude of bias, and the implications for the validity and individual-level generalisability of the adjusted estimates.

Response:

We agree and have substantially expanded the Strengths and Limitations subsection in the discussion. We have expanded the discussion of potential misclassification resulting from the use of ICD-10 and OPCS-4 codes. The revised manuscript acknowledges coding errors, variation in coding practices between centres and limited coding granularity. It also notes that procedures were not clinically adjudicated and that administrative procedure codes may not fully capture procedural intent or the sequence of previous treatments. Additionally, we have expanded the discussion of unmeasured confounding.

The revised manuscript identifies unavailable variables such as fracture pattern, wound status, American Society of Anesthesiologists classification, implant characteristics, and intraoperative details. These factors may influence both the procedure selected and subsequent mortality. Furthermore, we have also discussed the possible direction of residual confounding, but we acknowledge that it is not possible to reliably determine or quantify the magnitude of these opposing biases using the available administrative data.

We have also clarified the implications for validity and clinical generalisability. We note that the limitations primarily affect comparisons between case-mix-adjusted estimates rather than ascertainment of observed all-cause mortality, which was based on national mortality records. Therefore, we caution that the adjusted estimates should be interpreted as population-level standardised descriptions conditional on the measured covariates. They should not be regarded as estimates that fully account for clinical severity and procedural complexity or as individualised prognostic estimates.

Specifically, we have made the following revisions to our manuscript (lines refer to the marked version of the main document):

- Expanded discussion of ICD-10 and OPCS-4 administrative coding (lines 438–450)
- Expanded discussion of unmeasured confounding and its potential direction (lines 452–465)
- Clarified the interpretation of case-mix-adjusted estimates in the context of administrative data (lines 472–477)

- Clarification of individual clinical interpretation (lines 495–502 and 511–514)

Reviewer 2 Comments

We thank the reviewer for their positive assessment of the manuscript, its methodology and reporting, and for concluding that the findings are appropriately supported by the analysis and represent a useful contribution to orthopaedic epidemiology.

No specific revisions were requested in response to this review.

VERSION 2 - REVIEW

Reviewer 1

Name Yeh, Kuang-Ting

Affiliation Hualien Tzu Chi Hospital Buddhist Tzu Chi Medical Foundation, Department of Orthopedics

Date 13-Aug-2026

COI

The authors have revised their manuscript well according to the reviewers' suggestions. Thank you for your effort.
