# Supplementary material for: All-cause mortality following internal fixation for isolated fractures in non-polytrauma admissions: a comprehensive 10-year nationwide linked data retrospective cohort study
Source: BMJ Open. 2026 Sep 8;16(9):e122170. doi: 10.1136/bmjopen-2026-122170 (PMC13560970; doi:10.1136/bmjopen-2026-122170)
Supplement: Supplementary file 3 [file bmjopen-16-9-s001.docx]

Supplementary table A. – Procedure definition according to OPCS-v4 codes

| **Procedure** | **OPCS** | **Description** | **Admissions**  **(primary analysis)** |
| --- | --- | --- | --- |
| Primary open reduction | W19.2 | Primary open reduction of fracture of long bone and fixation using rigid nail | 52,492 ( 8.3) |
|  | W19.3 | Primary open reduction of fracture of long bone and fixation using flexible nail | 2,499 ( 0.4) |
|  | W19.4 | Primary open reduction of fracture of short bone and fixation using screw | 11,388 ( 1.8) |
|  | W19.5 | Primary open reduction of fragment of bone and fixation using screw | 9,938 ( 1.6) |
|  | W19.6 | Primary open reduction of fragment of bone and fixation using wire system | 3,969 ( 0.6) |
|  | W19.8 | Other specified [primary open reduction of fracture of bone and intramedullary fixation] | 33,518 ( 5.3) |
|  | W19.9 | Unspecified [primary open reduction of fracture of bone and intramedullary fixation] | 2,031 ( 0.3) |
|  | W20.1 | Primary open reduction of fracture of long bone and extramedullary fixation using plate | 171,679 (27.2) |
|  | W20.2 | Primary open reduction of fracture of long bone and extramedullary fixation using cerclage | 13,062 ( 2.1) |
|  | W20.3 | Primary open reduction of fracture of long bone and extramedullary fixation using suture | 1,731 ( 0.3) |
|  | W20.4 | Primary open reduction of fracture of long bone and complex extramedullary fixation | 776 ( 0.1) |
|  | W20.5 | Primary open reduction of fracture of ankle and extramedullary fixation | 19,624 ( 3.1) |
|  | W20.8 | Other specified [primary open reduction of fracture of bone and extramedullary fixation] | 13,385 ( 2.1) |
|  | W20.9 | Unspecified [primary open reduction of fracture of bone and extramedullary fixation] | 3,291 ( 0.5) |
| Secondary open reduction | W23.1 | Secondary open reduction of fracture of bone and intramedullary fixation | 9,786 ( 1.6) |
|  | W23.2 | Secondary open reduction of fracture of bone and extramedullary fixation  Includes:  Secondary open reduction of fracture of bone and tension band wiring | 39,055 ( 6.2) |
|  | W23.6 | Secondary open reduction of fracture of bone and internal fixation | 6,481 ( 1.0) |
| Closed reduction | W24.2 | Closed reduction of fracture of long bone and rigid internal fixation | 91,464 (14.5) |
|  | W24.3 | Closed reduction of fracture of long bone and flexible internal fixation | 2,189 ( 0.3) |
|  | W24.4 | Closed reduction of fracture of short bone and fixation using screw | 2,534 ( 0.4) |
|  | W24.5 | Closed reduction of fragment of bone and fixation using screw | 739 ( 0.1) |
|  | W24.6 | Closed reduction of fracture of bone and fixation using nail or screw | 118,300 (18.8) |
|  | W24.7 | Closed reduction of fracture of bone and fixation using plate | 3,131 ( 0.5) |
|  | W24.8 | Other specified [closed reduction of fracture of bone and internal fixation] | 3,367 ( 0.5) |
|  | W24.9 | Unspecified [closed reduction of fracture of bone and internal fixation] | 323 ( 0.1) |
| Other | W28.1 | Application of internal fixation to bone | 11,827 ( 1.9) |
|  | W28.2 | Adjustment to internal fixation of bone | 2,148 ( 0.3) |
|  | W28.9 | Unspecified [other internal fixation of bone] | 31 ( 0.0) |
