# Supplementary material for: All-cause mortality following internal fixation for isolated fractures in non-polytrauma admissions: a comprehensive 10-year nationwide linked data retrospective cohort study
Source: BMJ Open. 2026 Sep 8;16(9):e122170. doi: 10.1136/bmjopen-2026-122170 (PMC13560970; doi:10.1136/bmjopen-2026-122170)
Supplement: Supplementary file 4 [file bmjopen-16-9-s002.docx]

Supplementary table B - ICD10 codes used to define comorbidities

| Comorbidity | ICD-10 |
| --- | --- |
| Congestive heart failure | I09.9, I11.0, I13.0, I13.2, I25.5, I42.0, I42.5, I42.6, I42.7, I42.8,  I42.9, I43.X, I50.X, P29.0 |
| Cardiac dysrhythmias | I44.1, I44.2, I44.3, I45.6, I45.9, I47.X, I48.X, I49.X, R00.0, R00.1,  R00.8, T82.1, Z45.0, Z95.0 |
| Valvular disease | A52.0, I05.X, I06.X, I07.X, I08.X, I09.1, I09.8, I34.X, I35.X, I36.X,  I37.X, I38.X, I39.X, Q23.0, Q23.1, Q23.2, Q23.3, Z95.2, Z95.3, Z95.4 |
| Pulmonary circulation disorders | I26.X, I27.X, I28.0, I28.8, I28.9 |
| Peripheral vascular disease | I70.X, I71.X, I73.1, I73.8, I73.9, I77.1, I79.0, I79.2, K55.1, K55.8,  K55.9, Z95.8, Z95.9 |
| Systemic arterial hypertension (mild) | I10.X |
| Systemic arterial hypertension (Severe) | I11.X, I12.X, I13.X, I15.X |
| Paralysis | G04.1, G11.4, G80.1, G80.2, G81.X, G82.X, G83.0, G83.1, G83.2, G83.3,  G83.4, G83.9 |
| Neurodegenerative disorders | G10.X, G11.X, G12.X, G13.X, G20.X, G21.X, G22.X, G25.4, G25.5, G31.2,  G31.8, G31.9, G32.X, G35.X, G36.X, G37.X, G40.X, G41.X, G93.1, G93.4, R47.0, R56.X |
| Chronic pulmonary disease | I27.8, I27.9, J40.X, J41.X, J42.X, J43.X, J44.X, J45.X, J46.X, J47.X,  J60.X, J61.X, J62.X, J63.X, J64.X, J65.X, J66.X, J67.X, J68.4, J70.1, J70.3 |
| Diabetes mellitus (uncomplicated) | E10.0, E10.1, E10.9, E11.0, E11.1, E11.9, E12.0, E12.1, E12.9, E13.0,  E13.1, E13.9, E14.0, E14.1, E14.9 |
| Diabetes mellitus (complicated) | E10.2, E10.3, E10.4, E10.5, E10.6, E10.7, E10.8, E11.2, E11.3, E11.4,  E11.5, E11.6, E11.7, E11.8, E12.2, E12.3, E12.4, E12.5, E12.6, E12.7,  E12.8, E13.2, E13.3, E13.4, E13.5, E13.6, E13.7, E13.8, E14.2, E14.3,  E14.4, E14.5, E14.6, E14.7, E14.8 |
| Hypothyroidism | E00.X, E01.X, E02.X, E03.X, E89.0 |
| Chronic kidney disease | I12.0, I13.1, N18.X, N19.X, N25.0, Z49.0, Z49.1, Z49.2, Z94.0, Z199.2 |
| Liver disease | B18.X, I85.X, I86.4, I98.2, K70.X, K71.1, K71.3, K71.4, K71.5, K71.7,  K72.X, K73.X, K74.X, K76.0, K76.2, K76.3, K76.4, K76.5, K76.6, K76.7,  K76.8, K76.9, Z94.4 |
| Peptic ulcer disease | K25.7, K25.9, K26.7, K26.9, K27.7, K27.9, K28.7, K28.9 |
| AIDS/HIV | B20.X, B21.X, B22.X, B24.X |
| Lymphoma | C81.X, C82.X, C83.X, C84.X, C85.X, C88.X, C96.X, C90.0, C90.2 |
| Metastatic cancer | C77.X, C78.X, C79.X, C80.X |
| Solid tumour | C00.X, C01.X, C02.X, C03.X, C04.X, C05.X, C06.X, C07.X, C08.X, C09.X,  C10.X, C11.X, C12.X, C13.X, C14.X, C15.X, C16.X, C17.X, C18.X, C19.X,  C20.X, C21.X, C22.X, C23.X, C24.X, C25.X, C26.X, C30.X, C31.X, C32.X,  C33.X, C34.X, C37.X, C38.X, C39.X, C40.X, C41.X, C43.X, C45.X, C46.X,  C47.X, C48.X, C49.X, C50.X, C51.X, C52.X, C53.X, C54.X, C55.X, C56.X,  C57.X, C58.X, C60.X, C61.X, C62.X, C63.X, C64.X, C65.X, C66.X, C67.X,  C68.X, C69.X, C70.X, C71.X, C72.X, C73.X, C74.X, C75.X, C76.X, C97.X |
| Connective tissue disease | L94.0, L94.1, L94.3, M05.X, M06.X, M08.X, M12.0, M12.3, M30.X, M31.0,  M31.1, M31.2, M31.3, M32.X, M33.X, M34.X, M35.X, M45.X, M46.1, M46.8,  M46.9 |
| Coagulopathy | D65.X, D66.X, D67.X, D68.X, D69.1, D69.3, D69.4, D69.5, D69.6 |
| Obesity | E66.X |
| Weight loss | E40.X, E41.X, E42.X, E43.X, E44.X, E45.X, E46.X, R63.4, R64.X |
| Fluid and electrolyte disorders | E22.2, E86.X, E87.X |
| Blood loss anaemia | D50.0 |
| Deficiency anaemia | D50.8, D50.9, D51.X, D52.X, D53.X |
| Alcohol misuse | F10.X, E52.X, G62.1, I42.6, K29.2, K70.0, K70.3, K70.9, T51.X, Z50.2,  Z71.4, Z72.1 |
| Drug abuse | F11.X, F12.X, F13.X, F14.X, F15.X, F16.X, F18.X, F19.X, Z71.5, Z72.2 |
| Psychosis | F20.X, F22.X, F23.X, F24.X, F25.X, F28.X, F29.X, F30.2, F31.2, F31.5 |
| Depression | F20.4, F31.3, F31.4, F31.5, F32.X, F33.X, F34.1, F41.2, F43.2 |
| Dementia | F00.X, F01.X, F02.X, F03.X, F05.X, G3.0 |
| Delirium | F05.X |
| Osteoporosis | M80.X, M81.X, M82.X |
| Paget's disease | M88.X |
| Smoking/nicotine dependence | F17.2, O99.3, P04.2, P96.8, T65.2, Z57.3, Z71.6, Z72.X, Z77.2, Z87.8 |
| Transport accident | VX.X |
