# Supplementary material for: All-cause mortality following internal fixation for isolated fractures in non-polytrauma admissions: a comprehensive 10-year nationwide linked data retrospective cohort study
Source: BMJ Open. 2026 Sep 8;16(9):e122170. doi: 10.1136/bmjopen-2026-122170 (PMC13560970; doi:10.1136/bmjopen-2026-122170)
Supplement: Supplementary file 5 [file bmjopen-16-9-s003.docx]

Supplementary table C – Fracture region definition

| **Fracture region** | **OPCS – V4 code** | **ICD 10 code** |
| --- | --- | --- |
| Shoulder girdle | Clavicle (Z68.1); Acromion process of scapula (Z68.2); Coracoid process of scapula (Z68.3); Glenoid cavity of scapula (Z68.4); Scapula nec (Z68.5); Specified bone of shoulder girdle nec (Z68.8); Bone of shoulder girdle nec (Z68.9); Sternoclavicular joint (Z81.1); Acromioclavicular joint (Z81.2); Shoulder joint (Z81.4); Shoulder nec (Z89.1) | Clavicle (S42.0); Scapula (S42.1); Shoulder girdle unsp (S42.9) |
| Humerus | Head of humerus (Z69.1); Tuberosity of humerus (Z69.2); Neck of humerus (Z69.3); Mid shaft of humerus (Z69.4); Lateral condyle of humerus (Z69.5); Medial epicondyle of humerus (Z69.6); Lower end of humerus nec (Z69.7); Specified humerus nec (Z69.8); Humerus nec (Z69.9) | Upper humerus (S42.2); Shaft humerus (S42.3); Lower humerus (S42.4) |
| Radius | Head of radius (Z70.1); Neck of radius (Z70.2); Shaft of radius nec (Z70.3); Styloid process of radius (Z70.4); Lower end of radius nec (Z70.5); Specified radius nec (Z70.8); Radius nec (Z70.9); Radiocarpal joint (Z82.1) | Upper radius (S52.1); Shaft radius (S52.3); Lower radius (S52.5) |
| Ulna | Olecranon process of ulna (Z71.1); Shaft of ulna nec (Z71.2); Coronoid process of ulna (Z71.3); Head of ulna (Z71.4); Styloid process of ulna (Z71.5); Lower end of ulna nec (Z71.6); Specified ulna nec (Z71.8); Ulna nec (Z71.9) | Upper ulna (S52.0); Shaft ulna (S52.2) |
| Hand | Scaphoid bone (Z72.2); Lunate (Z72.3); Carpal bone nec (Z72.4); Trapezium (Z72.5); First metacarpal (Z73.1); Metacarpal nec (Z73.2); Phalanx of thumb (Z73.3); Phalanx of finger (Z73.4); Specified bone of hand nec (Z73.8); Bone of hand nec (Z73.9); Intercarpal joint (Z82.2); Carpometacarpal joint of thumb (Z82.3); Carpometacarpal joint of finger (Z82.4); Scapholunate joint (Z82.5); Lunotriquetral joint (Z82.6); Scaphotrapeziotrapezoidal joint (Z82.7); Metacarpophalangeal joint of thumb (Z83.1); Metacarpophalangeal joint of finger (Z83.2); Interphalangeal joint of thumb (Z83.3); Proximal interphalangeal joint of finger (Z83.4); Distal interphalangeal joint of finger (Z83.5); Interphalangeal joint of finger nec (Z83.6); Specified joint of finger nec (Z83.8); Joint of finger nec (Z83.9); Hand nec (Z89.4); Thumb nec (Z89.5); Finger nec (Z89.6) | Navicular bone (S62.0); Other carpal (S62.1); First metacarpal (S62.2); Other metacarpal (S62.3); Thumb (S62.5); Hand finger (S62.6) |
| Arm (other) | Specified bone of arm or wrist nec (Z72.8); Bone of arm or wrist nec (Z72.9); Glenohumeral joint (Z81.3); Elbow joint (Z81.5); Superior radioulnar joint (Z81.6); Inferior radioulnar joint (Z81.7); Specified joint of shoulder girdle or arm nec (Z81.8); Joint of shoulder girdle or arm nec (Z81.9); Upper arm nec (Z89.2); Forearm nec (Z89.3); Specified arm region nec (Z89.8); Arm nec (Z89.9); Specified joint of wrist or hand nec (Z82.8); Joint of wrist or hand nec (Z82.9) | Wrist hand unsp (S62.8); Other upr arm (S42.8); Other forearm (S52.8); Forearm unsp (S52.9) |
| Pelvis | Body of sacrum (Z75.1); Ala of sacrum (Z75.2); Wing of ilium (Z75.3); Ischium (Z75.4); Ramus of pubis (Z75.5); Acetabulum (Z75.6); Coccyx (Z75.7); Specified bone of pelvis nec (Z75.8); Bone of pelvis nec (Z75.9); Sacroiliac joint (Z84.1); Pubic symphysis (Z84.2) | Sacrum (S32.1); Coccyx (S32.2); Ilium (S32.3); Acetabulum (S32.4); Pubis (S32.5) |
| Femur | Hip joint (Z84.3); Hip nec (Z90.2); Head of femur (Z76.1); Neck of femur (Z76.2); Trochanter of femur (Z76.3); Shaft of femur (Z76.4); Lower end of femur nec (Z76.5); Trochlear surface of femur (Z76.6); Specified femur nec (Z76.8); Femur nec (Z76.9) | Neck femur (S72.0); Pertrochanteric (S72.1); Subtrochanteric (S72.2); Shaft femur (S72.3); Lower femur (S72.4); Other femur (S72.8); Femur unsp (S72.9) |
| Tibia | Condyle of tibia (Z77.1); Shaft of tibia nec (Z77.2); Medial malleolus (Z77.3); Upper end of tibia nec (Z77.4); Lower end of tibia nec (Z77.5); Tuberosity of tibia (Z77.6); Specified tibia nec (Z77.8); Tibia nec (Z77.9) | Upper tibia (S82.1); Shaft tibia (S82.2); Lower tibia (S82.3); Medial malleolus (S82.5) |
| Fibula | Head of fibula (Z78.2); Shaft of fibula nec (Z78.3); Lateral malleolus (Z78.4); Lower end of fibula nec (Z78.5); Fibula nec (Z78.6) | Fibula alone (S82.4); Lateral malleolus (S82.6) |
| Leg (other) | Patella (Z78.7); Specified bone of lower leg nec (Z78.8); Bone of lower leg nec (Z78.9); Patellofemoral joint (Z84.4); Tibiofemoral joint (Z84.5); Knee joint (Z84.6); Specified joint of pelvis or upper leg nec (Z84.8); Joint of pelvis or upper leg nec (Z84.9); Upper tibiofibular joint (Z85.1); Lower tibiofibular joint (Z85.2); Buttock nec (Z90.1); Upper leg nec (Z90.3); Lower leg nec (Z90.4); Specified leg region nec (Z90.8); Leg region nec (Z90.9) | Patella (S82.0); Other lower leg (S82.8); Lower leg unsp (S82.9) |
| Tarsus | Talus (Z79.1); Os calcis (Z79.2); Navicular bone of foot (Z79.3); Cuboid bone (Z79.4); Cuneiform bone (Z79.5); Specified bone of tarsus nec (Z79.8); Bone of tarsus nec (Z79.9); Talocalcaneal joint (Z85.3); Talonavicular joint (Z85.4); Calcaneocuboid joint (Z85.5); Ankle joint (Z85.6); Specified joint of lower leg or tarsus nec (Z85.8); Joint of lower leg or tarsus nec (Z85.9); Midtarsal joint (Z86.1); Intertarsal joint (Z86.2); Tarsometatarsal joint (Z86.3) | Calcaneus (S92.0); Talus (S92.1); Other tarsal (S92.2) |
| Foot | First metatarsal (Z80.1); Metatarsal nec (Z80.2); Phalanx of great toe (Z80.3); Phalanx of toe nec (Z80.4); Specified bone of foot nec (Z80.8); Bone of foot nec (Z80.9); Metatarsophalangeal joint of great toe (Z86.4); Metatarsophalangeal joint of toe nec (Z86.5); Interphalangeal joint of toe nec (Z86.6); Interphalangeal joint of great toe (Z86.7); Specified joint of foot nec (Z86.8); Joint of foot nec (Z86.9); Foot nec (Z90.5); Great toe nec (Z90.6); Toe nec (Z90.7) | Metatarsal (S92.3); Great toe (S92.4); Other toe (S92.5); Foot unsp (S92.9) |
| Other | Frontal bone (Z63.1); Parietal bone (Z63.2); Temporal bone (Z63.3); Occipital bone (Z63.4); Specified bone of cranium nec (Z63.8); Bone of cranium nec (Z63.9); Nasoethmoid complex of bones (Z64.1); Nasal bone (Z64.2); Zygomatic complex of bones (Z64.3); Maxilla (Z64.4); Specified bone of face nec (Z64.8); Bone of face nec (Z64.9); Mandible (Z65.1); Temporomandibular joint (Z65.2); Specified jaw nec (Z65.8); Jaw nec (Z65.9); Atlas (Z66.1); Axis bone (Z66.2); Cervical vertebra (Z66.3); Thoracic vertebra (Z66.4); Lumbar vertebra (Z66.5); Specified vertebra nec (Z66.8); Vertebra nec (Z66.9); Atlanto occipital joint (Z67.1); Atlantoaxial joint (Z67.2); Cervical intervertebral joint (Z67.3); Thoracic intervertebral joint (Z67.4); Lumbar intervertebral joint (Z67.5); Lumbosacral joint (Z67.6); Sacrococcygeal joint (Z67.7); Specified intervertebral joint nec (Z67.8); Intervertebral joint nec (Z67.9); Manubrium sterni (Z74.1); Sternum nec (Z74.2); First rib (Z74.3); Second to tenth rib (Z74.4); Eleventh or twelfth rib (Z74.5); Rib nec (Z74.6); Specified rib cage nec (Z74.8); Rib cage nec (Z74.9); Bone nec (Z87.1); Ligament of joint (Z87.2); Capsule of joint (Z87.3); Joint nec (Z87.4); Specified part of musculoskeletal system nec (Z87.8); Musculoskeletal system nec (Z87.9); Hyoid (Z88.1); Epiglottis (Z88.2); Glottis (Z88.3); Supraglottis (Z88.4); Subglottis (Z88.5); Specified respiratory tract nec (Z88.8); Respiratory tract nec (Z88.9) | Skull vault (S02.0); Skull base (S02.1); Nasal bones (S02.2); Orbital floor (S02.3); Malar maxillar (S02.4); Mandible (S02.6); Skull others (S02.8); Skull unsp (S02.9); First cervical (S12.0); Second cervical (S12.1); Other cervical (S12.2); Neck other (S12.8); Neck unsp (S12.9); Thorax vertebra (S22.0); Thorax spine (S22.1); Thorax sternum (S22.2); Thorax rib (S22.3); Other thorax (S22.8); Thorax unsp (S22.9); Lumbar vertebra (S32.0); Lumbosacrum unsp (S32.8) |
| Multiple | Shafts of radius and ulna in combination (Z72.1); Shafts of tibia and fibula in combination (Z78.1); Multiple digits of hand nec (Z89.7); Multiple metacarpals (Z73.5); Multiple metatarsals (Z80.5) | Multiple skull (S02.7); Multiple cervical (S12.7); Multiple ribs (S22.4); Flail chest (S22.5); Multiple lumbosacrum (S32.7); Multiple upr arm (S42.7); Shafts ulna radius (S52.4); Lower ulna radius (S52.6); Multiple forearm (S52.7); Multiple metacarpal (S62.4); Multiple hand fingers (S62.7); Multiple femur (S72.7); Multiple lower leg (S82.7); Multiple foot (S92.7); Multiple foot (S92.7); Multiregion head neck (T02.0); Multiregion thorax lumbosacrum (T02.1); Multiregion one upper limb (T02.2); Multiregion one lower limb (T02.3); Multiregion both upper limb (T02.4); Multiregion both lower limb (T02.5); Multiregion upper lower limb (T02.6); Multiregion thorax lumbosacrum limb (T02.7); Multiregion other combinations (T02.8); Multiregion unsp (T02.9) |
