# Supplementary material for: All-cause mortality following internal fixation for isolated fractures in non-polytrauma admissions: a comprehensive 10-year nationwide linked data retrospective cohort study
Source: BMJ Open. 2026 Sep 8;16(9):e122170. doi: 10.1136/bmjopen-2026-122170 (PMC13560970; doi:10.1136/bmjopen-2026-122170)
Supplement: Supplementary file 6 [file bmjopen-16-9-s004.docx]

Supplementary table D – Cause of death according to ICD-10

| Cause of death | ICD-10 |
| --- | --- |
| Surgical complications | I97.X, K91.3, K91.8, K91.9, T81.0, T81.1, T81.2, T81.3, T81.4, T81.7, T81.8, T81.9, T84.X, T88.9, T98.3, Y60.0, Y83.2, Y83.3, Y83.4, Y83.6, Y83.8, Y83.9, Y88.3, Z47.X |
| Cardiovascular deaths |  |
| Myocardial infarction | I25.X |
| Heart failure | I11.X |
| Cerebrovascular | I60.X I61.X I62.X I63.X I64.X I65.X I66.X I67.X I68.X I69.X |
| Intestinal infarction | K55.X |
| Deaths due to infection |  |
| Sepsis | A41.X |
| Pneumonia | J12.X J13.X J14.X J15.X J16.X J17.X J18.X |
| Respiratory deaths |  |
| Pulmonary embolism | I26.X |
| Respiratory failure | I27.X |
| Malignancy | C00.X C01.X C02.X C03.X C04.X C05.X C06.X C07.X C08.X C09.X C10.X C11.X C12.X C13.X C14.X C15.X C16.X C17.X C18.X C19.X C20.X C21.X C22.X C23.X C24.X C25.X C26.X C27.X C28.X C29.X C30.X C31.X C32.X C33.X C34.X C35.X C36.X C37.X C38.X C39.X C40.X C41.X C42.X C43.X C44.X C45.X C46.X C47.X C48.X C49.X C50.X C51.X C52.X C53.X C54.X C55.X C56.X C57.X C58.X C59.X C60.X C61.X C62.X C63.X C64.X C65.X C66.X C67.X C68.X C69.X C70.X C71.X C72.X C73.X C74.X C75.X C76.X C77.X C78.X C79.X C80.X C81.X C82.X C83.X C84.X C85.X C86.X C87.X C88.X C89.X C90.X C91.X C92.X C93.X C94.X C95.X C96.X C97.X |
