# Supplementary material for: All-cause mortality following internal fixation for isolated fractures in non-polytrauma admissions: a comprehensive 10-year nationwide linked data retrospective cohort study
Source: BMJ Open. 2026 Sep 8;16(9):e122170. doi: 10.1136/bmjopen-2026-122170 (PMC13560970; doi:10.1136/bmjopen-2026-122170)
Supplement: Supplementary file 7 [file bmjopen-16-9-s005.docx]

Supplementary table E .- Missing data

|  | **Overall** | **Primary open reduction** | **Secondary open reduction** | **Closed reduction** | **Other internal fixation** |
| --- | --- | --- | --- | --- | --- |
| Female (missing), n (%) | 24 (0.0) | 15 (0.0) | 1 (0.0) | 6 (0.0) | 2 (0.0) |
| Area of residence (missing), n (%) | 6,400 (1.0) | 3,713 (1.1) | 517 (0.9) | 2,048 (0.9) | 122 (0.9) |
| Post-procedure stay in days (missing), n (%) | 981 (0.2) | 293 (0.1) | 10 (0.0) | 665 (0.3) | 13 (0.1) |
| Post-procedure stay >28 days (missing), n (%) | 981 (0.2) | 293 (0.1) | 10 (0.0) | 665 (0.3) | 13 (0.1) |
