# Supplementary material for: All-cause mortality following internal fixation for isolated fractures in non-polytrauma admissions: a comprehensive 10-year nationwide linked data retrospective cohort study
Source: BMJ Open. 2026 Sep 8;16(9):e122170. doi: 10.1136/bmjopen-2026-122170 (PMC13560970; doi:10.1136/bmjopen-2026-122170)
Supplement: Supplementary file 8 [file bmjopen-16-9-s006.docx]

Supplementary table F.- Characteristics by fracture region

|  | **Shoulder girdle** | **Humerus** | **Radius** | **Ulna** | **Hand** | **Arm (other)** | **Pelvis** | **Femur** | **Tibia** | **Fibula** | **Leg (other)** | **Tarsus** | **Foot** | **Other** |
| --- | --- | --- | --- | --- | --- | --- | --- | --- | --- | --- | --- | --- | --- | --- |
| Admissions | 31,553 | 43,205 | 108,690 | 24,576 | 94,327 | 632 | 2,295 | 217,706 | 47,733 | 27,212 | 17,203 | 6,344 | 8,539 | 743 |
| Female, n (%) | 7,510 (23.8) | 26,430 (61.2) | 77,108 (70.9) | 12,635 (51.4) | 21,426 (22.7) | 371 (58.8) | 790 (34.4) | 150,878 (69.3) | 20,044 (42.0) | 12,560 (46.2) | 10,406 (60.5) | 2,182 (34.4) | 4,190 (49.1) | 196 (26.4) |
| Age in years, mean ± SD | 40.0 ± 14.6 | 57.6 ± 18.0 | 55.5 ± 17.0 | 53.7 ± 20.0 | 36.2 ± 15.3 | 51.4 ± 18.2 | 55.6 ± 19.8 | 79.3 ± 14.1 | 45.6 ± 18.1 | 44.8 ± 16.9 | 51.4 ± 19.0 | 43.4 ± 16.0 | 43.6 ± 16.4 | 48.1 ± 18.4 |
| Ethnicity, n (%) |  |  |  |  |  |  |  |  |  |  |  |  |  |  |
| White | 26,202 (83.0) | 36,580 (84.7) | 91,337 (84.0) | 20,531 (83.5) | 71,394 (75.7) | 523 (82.8) | 1,579 (68.8) | 191,505 (88.0) | 37,479 (78.5) | 21,910 (80.5) | 13,776 (80.1) | 5,091 (80.2) | 6,875 (80.5) | 613 (82.5) |
| Black | 219 (0.7) | 399 (0.9) | 885 (0.8) | 254 (1.0) | 2,147 (2.3) | 6 (0.9) | 25 (1.1) | 946 (0.4) | 855 (1.8) | 757 (2.8) | 376 (2.2) | 98 (1.5) | 161 (1.9) | 3 (0.4) |
| Asian | 620 (2.0) | 1,129 (2.6) | 2,874 (2.6) | 649 (2.6) | 4,259 (4.5) | 18 (2.8) | 54 (2.4) | 3,772 (1.7) | 1,616 (3.4) | 703 (2.6) | 584 (3.4) | 204 (3.2) | 316 (3.7) | 8 (1.1) |
| Mixed | 241 (0.8) | 234 (0.5) | 500 (0.5) | 134 (0.5) | 1,233 (1.3) | 5 (0.8) | 18 (0.8) | 385 (0.2) | 391 (0.8) | 304 (1.1) | 145 (0.8) | 57 (0.9) | 93 (1.1) | 7 (0.9) |
| Other | 664 (2.1) | 710 (1.6) | 1,847 (1.7) | 492 (2.0) | 2,605 (2.8) | 9 (1.4) | 69 (3.0) | 2,219 (1.0) | 1,301 (2.7) | 594 (2.2) | 375 (2.2) | 149 (2.3) | 188 (2.2) | 10 (1.3) |
| Unknown | 3,607 (11.4) | 4,153 (9.6) | 11,247 (10.3) | 2,516 (10.2) | 12,689 (13.5) | 71 (11.2) | 550 (24.0) | 18,879 (8.7) | 6,091 (12.8) | 2,944 (10.8) | 1,947 (11.3) | 745 (11.7) | 906 (10.6) | 102 (13.7) |
| Area of residence, n (%) |  |  |  |  |  |  |  |  |  |  |  |  |  |  |
| Urban | 24,095 (77.1) | 33,990 (79.4) | 84,505 (78.4) | 19,135 (78.8) | 76,178 (81.6) | 481 (77.2) | 1,675 (74.4) | 166,065 (77.0) | 37,019 (78.8) | 21,205 (78.8) | 13,096 (77.1) | 4,967 (79.4) | 6,777 (80.1) | 558 (76.5) |
| Town | 3,032 (9.7) | 4,215 (9.9) | 10,671 (9.9) | 2,270 (9.3) | 7,574 (8.1) | 69 (11.1) | 239 (10.6) | 23,869 (11.1) | 4,348 (9.3) | 2,524 (9.4) | 1,690 (10.0) | 565 (9.0) | 794 (9.4) | 73 (10.0) |
| Rural | 4,140 (13.2) | 4,582 (10.7) | 12,577 (11.7) | 2,888 (11.9) | 9,625 (10.3) | 73 (11.7) | 338 (15.0) | 25,796 (12.0) | 5,590 (11.9) | 3,170 (11.8) | 2,189 (12.9) | 720 (11.5) | 893 (10.6) | 98 (13.4) |
| Emergency admission, n (%) | 9,671 (30.7) | 20,144 (46.6) | 48,848 (44.9) | 13,668 (55.6) | 29,854 (31.6) | 212 (33.5) | 1,984 (86.4) | 212,153 (97.4) | 38,914 (81.5) | 19,159 (70.4) | 13,692 (79.6) | 3,182 (50.2) | 3,430 (40.2) | 263 (35.4) |
| Dementia, n (%) | 44 (0.1) | 886 (2.1) | 735 (0.7) | 406 (1.7) | 172 (0.2) | 3 (0.5) | 107 (4.7) | 59,542 (27.3) | 580 (1.2) | 109 (0.4) | 228 (1.3) | 17 (0.3) | 35 (0.4) | 10 (1.3) |
| Osteoporosis, n (%) | 238 (0.8) | 2,272 (5.3) | 4,762 (4.4) | 1,463 (6.0) | 318 (0.3) | 28 (4.4) | 152 (6.6) | 42,028 (19.3) | 1,397 (2.9) | 313 (1.2) | 608 (3.5) | 108 (1.7) | 90 (1.1) | 18 (2.4) |
| Congestive heart failure, n (%) | 52 (0.2) | 572 (1.3) | 564 (0.5) | 205 (0.8) | 130 (0.1) | 3 (0.5) | 36 (1.6) | 21,840 (10.0) | 461 (1.0) | 137 (0.5) | 174 (1.0) | 20 (0.3) | 41 (0.5) | 13 (1.7) |
| Cardiac dysrhythmias, n (%) | 374 (1.2) | 2,462 (5.7) | 3,086 (2.8) | 1,088 (4.4) | 839 (0.9) | 16 (2.5) | 199 (8.7) | 52,410 (24.1) | 1,622 (3.4) | 636 (2.3) | 688 (4.0) | 119 (1.9) | 168 (2.0) | 51 (6.9) |
| Systemic arterial hypertension, n (%) | 1,988 (6.3) | 12,195 (28.2) | 21,647 (19.9) | 4,894 (19.9) | 5,264 (5.6) | 101 (16.0) | 517 (22.5) | 109,569 (50.3) | 7,046 (14.8) | 4,088 (15.0) | 3,650 (21.2) | 748 (11.8) | 1,101 (12.9) | 181 (24.4) |
| Chronic pulmonary disease, n (%) | 2,927 (9.3) | 6,331 (14.7) | 13,227 (12.2) | 3,001 (12.2) | 8,218 (8.7) | 79 (12.5) | 285 (12.4) | 45,156 (20.7) | 5,441 (11.4) | 3,170 (11.6) | 2,154 (12.5) | 716 (11.3) | 1,057 (12.4) | 141 (19.0) |
| Diabetes mellitus, n (%) | 736 (2.3) | 4,406 (10.2) | 5,136 (4.7) | 1,372 (5.6) | 2,373 (2.5) | 18 (2.8) | 199 (8.7) | 36,890 (16.9) | 3,013 (6.3) | 1,549 (5.7) | 1,385 (8.1) | 312 (4.9) | 463 (5.4) | 95 (12.8) |
| Liver disease, n (%) | 151 (0.5) | 710 (1.6) | 649 (0.6) | 244 (1.0) | 290 (0.3) | 7 (1.1) | 47 (2.0) | 4,386 (2.0) | 507 (1.1) | 142 (0.5) | 123 (0.7) | 43 (0.7) | 52 (0.6) | 18 (2.4) |
| Lymphoma, n (%) | 20 (0.1) | 560 (1.3) | 130 (0.1) | 38 (0.2) | 33 (0.0) | 1 (0.2) | 5 (0.2) | 2,171 (1.0) | 82 (0.2) | 19 (0.1) | 33 (0.2) | 0 (0.0) | 8 (0.1) | 4 (0.5) |
| Metastatic cancer, n (%) | 17 (0.1) | 1,829 (4.2) | 164 (0.2) | 72 (0.3) | 25 (0.0) | 2 (0.3) | 21 (0.9) | 9,066 (4.2) | 261 (0.5) | 39 (0.1) | 33 (0.2) | 5 (0.1) | 12 (0.1) | 13 (1.7) |
| Solid tumour, n (%) | 55 (0.2) | 1,638 (3.8) | 510 (0.5) | 180 (0.7) | 156 (0.2) | 2 (0.3) | 43 (1.9) | 13,260 (6.1) | 362 (0.8) | 132 (0.5) | 124 (0.7) | 14 (0.2) | 21 (0.2) | 14 (1.9) |
| Elixhauser comorbidity index, median [IQR] | 0 [0, 0] | 0 [0, 3] | 0 [0, 0] | 0 [0, 0] | 0 [0, 0] | 0 [0, 0] | 0 [0, 3] | 4 [0, 9] | 0 [0, 0] | 0 [0, 0] | 0 [0, 0] | 0 [0, 0] | 0 [0, 0] | 0 [0, 3] |
| Hospital frailty score, median [IQR] | 2 [2, 3] | 3 [2, 5] | 1 [0, 2] | 1 [0, 2] | 0 [0, 1] | 1 [0, 2] | 3 [1, 5] | 8 [4, 13] | 1 [0, 2] | 1 [0, 2] | 1 [0, 3] | 0 [0, 2] | 0 [0, 1] | 0 [0, 2] |
| Abbreviated injury score (highest), median [IQR] | 0 [0, 0] | 0 [0, 0] | 0 [0, 0] | 0 [0, 0] | 0 [0, 0] | 0 [0, 0] | 0 [0, 0] | 0 [0, 0] | 0 [0, 0] | 0 [0, 0] | 0 [0, 0] | 0 [0, 0] | 0 [0, 0] | 0 [0, 0] |
| Injury severity score, median [IQR] | 0 [0, 0] | 0 [0, 0] | 0 [0, 0] | 0 [0, 0] | 0 [0, 0] | 0 [0, 0] | 0 [0, 0] | 0 [0, 0] | 0 [0, 0] | 0 [0, 0] | 0 [0, 0] | 0 [0, 0] | 0 [0, 0] | 0 [0, 0] |
| Injury severity score classification, n (%) |  |  |  |  |  |  |  |  |  |  |  |  |  |  |
| < 9 | 31,451 (99.7) | 42,843 (99.2) | 108,450 (99.8) | 24,257 (98.7) | 94,020 (99.7) | 631 (99.8) | 2,220 (96.7) | 212,935 (97.8) | 47,545 (99.6) | 27,181 (99.9) | 17,167 (99.8) | 6,332 (99.8) | 8,520 (99.8) | 739 (99.5) |
| 9 to 15 | 76 (0.2) | 320 (0.7) | 168 (0.2) | 276 (1.1) | 87 (0.1) | 0 (0.0) | 33 (1.4) | 4,302 (2.0) | 124 (0.3) | 28 (0.1) | 19 (0.1) | 6 (0.1) | 16 (0.2) | 2 (0.3) |
| 16 to 25 | 26 (0.1) | 33 (0.1) | 69 (0.1) | 41 (0.2) | 209 (0.2) | 1 (0.2) | 36 (1.6) | 441 (0.2) | 57 (0.1) | 2 (0.0) | 17 (0.1) | 6 (0.1) | 3 (0.0) | 2 (0.3) |
| 26+ | 0 (0.0) | 9 (0.0) | 3 (0.0) | 2 (0.0) | 11 (0.0) | 0 (0.0) | 6 (0.3) | 28 (0.0) | 7 (0.0) | 1 (0.0) | 0 (0.0) | 0 (0.0) | 0 (0.0) | 0 (0.0) |
| Admission to procedure in days, median [IQR] | 0 [0, 0] | 0 [0, 1] | 0 [0, 0] | 0 [0, 1] | 0 [0, 0] | 0 [0, 0] | 4 [1, 7] | 1 [0, 2] | 1 [0, 2] | 0 [0, 1] | 0 [0, 1] | 0 [0, 1] | 0 [0, 0] | 0 [0, 1] |
| Previous fixation procedures, median [IQR] | 0 [0, 0] | 0 [0, 0] | 0 [0, 0] | 0 [0, 0] | 0 [0, 0] | 50 (7.9) | 0 [0, 0] | 0 [0, 0] | 0 [0, 0] | 0 [0, 0] | 0 [0, 0] | 0 [0, 0] | 0 [0, 0] | 0 [0, 0] |
| Previous fixation procedures (cat), n (%) |  |  |  |  |  |  |  |  |  |  |  |  |  |  |
| 0 | 29,896 (94.7) | 40,677 (94.1) | 104,896 (96.5) | 22,805 (92.8) | 90,721 (96.2) | 582 (92.1) | 2,167 (94.4) | 204,077 (93.7) | 45,348 (95.0) | 26,555 (97.6) | 16,692 (97.0) | 5,916 (93.3) | 8,256 (96.7) | 703 (94.6) |
| 1 | 1,544 (4.9) | 2,259 (5.2) | 3,623 (3.3) | 1,618 (6.6) | 3,441 (3.6) | 50 (7.9) | 115 (5.0) | 12,668 (5.8) | 2,208 (4.6) | 638 (2.3) | 495 (2.9) | 407 (6.4) | 265 (3.1) | 37 (5.0) |
| 2 | 98 (0.3) | 236 (0.5) | 160 (0.1) | 124 (0.5) | 154 (0.2) | 0 (0.0) | 12 (0.5) | 821 (0.4) | 162 (0.3) | 19 (0.1) | 14 (0.1) | 21 (0.3) | 17 (0.2) | 3 (0.4) |
| 3 | 14 (0.0) | 28 (0.1) | 10 (0.0) | 23 (0.1) | 10 (0.0) | 0 (0.0) | 1 (0.0) | 100 (0.0) | 13 (0.0) | 0 (0.0) | 2 (0.0) | 0 (0.0) | 1 (0.0) | 0 (0.0) |
| 4+ | 1 (0.0) | 5 (0.0) | 1 (0.0) | 6 (0.0) | 1 (0.0) | 0 (0.0) | 0 (0.0) | 40 (0.0) | 2 (0.0) | 0 (0.0) | 0 (0.0) | 0 (0.0) | 0 (0.0) | 0 (0.0) |
| Post-procedure stay in days, median [IQR] | 0 [0, 1] | 1 [1, 2] | 0 [0, 1] | 1 [0, 1] | 0 [0, 0] | 0 [0, 1] | 8 [5, 16] | 14 [8, 26] | 2 [1, 5] | 1 [1, 2] | 2 [1, 4] | 1 [1, 2] | 0 [0, 1] | 2 [1, 4] |
| Post-procedure stay >28 days, n (%) | 30 (0.1) | 853 (2.0) | 260 (0.2) | 223 (0.9) | 43 (0.0) | 0 (0.0) | 247 (10.8) | 49,855 (23.0) | 1,470 (3.1) | 263 (1.0) | 475 (2.8) | 54 (0.9) | 40 (0.5) | 22 (3.0) |
| Discharge destination, n (%) |  |  |  |  |  |  |  |  |  |  |  |  |  |  |
| Home | 31,368 (99.4) | 41,595 (96.3) | 107,699 (99.1) | 24,063 (97.9) | 93,796 (99.4) | 623 (98.6) | 2,024 (88.2) | 152,945 (70.3) | 45,513 (95.3) | 26,591 (97.7) | 16,483 (95.8) | 6,224 (98.1) | 8,467 (99.2) | 719 (96.8) |
| Temporary | 89 (0.3) | 435 (1.0) | 408 (0.4) | 180 (0.7) | 205 (0.2) | 5 (0.8) | 71 (3.1) | 12,417 (5.7) | 808 (1.7) | 261 (1.0) | 257 (1.5) | 49 (0.8) | 26 (0.3) | 4 (0.5) |
| Care home | 27 (0.1) | 503 (1.2) | 238 (0.2) | 152 (0.6) | 39 (0.0) | 2 (0.3) | 78 (3.4) | 25,108 (11.5) | 602 (1.3) | 157 (0.6) | 228 (1.3) | 21 (0.3) | 17 (0.2) | 6 (0.8) |
| Other | 43 (0.1) | 314 (0.7) | 197 (0.2) | 117 (0.5) | 155 (0.2) | 2 (0.3) | 86 (3.7) | 10,711 (4.9) | 515 (1.1) | 131 (0.5) | 169 (1.0) | 33 (0.5) | 18 (0.2) | 7 (0.9) |
| Death | 6 (0.0) | 242 (0.6) | 33 (0.0) | 33 (0.1) | 10 (0.0) | 0 (0.0) | 28 (1.2) | 14,053 (6.5) | 153 (0.3) | 23 (0.1) | 27 (0.2) | 2 (0.0) | 6 (0.1) | 5 (0.7) |
| Unknown | 20 (0.1) | 116 (0.3) | 115 (0.1) | 31 (0.1) | 122 (0.1) | 0 (0.0) | 8 (0.3) | 2,472 (1.1) | 142 (0.3) | 49 (0.2) | 39 (0.2) | 15 (0.2) | 5 (0.1) | 2 (0.3) |
