# Supplementary material for: All-cause mortality following internal fixation for isolated fractures in non-polytrauma admissions: a comprehensive 10-year nationwide linked data retrospective cohort study
Source: BMJ Open. 2026 Sep 8;16(9):e122170. doi: 10.1136/bmjopen-2026-122170 (PMC13560970; doi:10.1136/bmjopen-2026-122170)
Supplement: Supplementary file 9 [file bmjopen-16-9-s007.docx]

Table G.- Crude and adjusted post-operative mortality rates by fracture region

| **Fracture region** | **Admissions** | **Within 30 days** | | **Within 90 days** | | **Within 365 days** | |
| --- | --- | --- | --- | --- | --- | --- | --- |
|  |  | **Crude** | **Adjusted*** | **Crude** | **Adjusted*** | **Crude** | **Adjusted*** |
| Shoulder girdle | 31,469 | 0.02% [0.01, 0.04] | 0.27% [0.27, 0.27] | 0.09% [0.06, 0.12] | 1.42% [1.41, 1.42] | 0.37% [0.30, 0.44] | 4.33% [4.31, 4.36] |
| Humerus | 40,523 | **0.36% [0.31, 0.42]** | 1.13% [1.12, 1.13] | **0.96% [0.87, 1.06]** | 3.07% [3.06, 3.08] | 2.86% [2.70, 3.02] | 7.20% [7.17, 7.23] |
| Radius | 107,995 | 0.06% [0.05, 0.08] | 0.36% [0.36, 0.37] | 0.18% [0.16, 0.21] | 1.16% [1.15, 1.17] | 0.79% [0.74, 0.85] | 3.98% [3.97, 3.99] |
| Ulna | 24,343 | 0.16% [0.12, 0.22] | 0.59% [0.59, 0.60] | 0.44% [0.36, 0.53] | 1.69% [1.68, 1.70] | 1.63% [1.48, 1.80] | 5.14% [5.12, 5.16] |
| Hand | 94,134 | 0.02% [0.01, 0.03] | 0.27% [0.27, 0.27] | 0.07% [0.05, 0.08] | 1.20% [1.19, 1.21] | 0.27% [0.24, 0.31] | 3.75% [3.73, 3.77] |
| Arm (other) | 628 | 0.16% [0.03, 0.90] | 0.99% [0.99, 1.00] | 0.32% [0.09, 1.15] | 2.14% [2.13, 2.15] | 1.27% [0.65, 2.49] | 6.21% [6.19, 6.23] |
| Pelvis | 2,243 | 0.76% [0.47, 1.21] | 1.20% [1.19, 1.20] | 2.05% [1.54, 2.72] | 3.48% [3.46, 3.49] | 4.55% [3.76, 5.49] | 7.33% [7.30, 7.35] |
| Femur | 200,130 | **6.10% [6.00, 6.21]** | 2.31% [2.30, 2.32] | **12.75% [12.61, 12.90]** | 4.81% [4.78, 4.82] | 24.02% [23.84, 24.21] | 9.43% [9.40, 9.47] |
| Tibia | 47,220 | **0.28% [0.24, 0.34]** | 1.23% [1.22, 1.24] | **0.63% [0.56, 0.71]** | 2.98% [2.96, 2.99] | 1.70% [1.59, 1.82] | 6.90% [6.88, 6.93] |
| Fibula | 27,050 | 0.10% [0.07, 0.15] | 0.67% [0.67, 0.67] | 0.23% [0.18, 0.29] | 1.75% [1.74, 1.75] | 0.64% [0.55, 0.74] | 4.19% [4.17, 4.21] |
| Leg (other) | 17,039 | 0.18% [0.12, 0.25] | 0.65% [0.65, 0.66] | 0.40% [0.31, 0.51] | 1.66% [1.65, 1.67] | 1.33% [1.17, 1.52] | 4.77% [4.76, 4.79] |
| Tarsus | 6,329 | 0.03% [0.01, 0.12] | 0.29% [0.29, 0.29] | 0.09% [0.04, 0.21] | 1.04% [1.03, 1.05] | 0.55% [0.40, 0.77] | 4.49% [4.47, 4.51] |
| Foot | 8,503 | 0.09% [0.05, 0.19] | 0.83% [0.82, 0.83] | 0.16% [0.10, 0.28] | 1.67% [1.66, 1.68] | 0.61% [0.47, 0.80] | 4.79% [4.76, 4.81] |
| Other | 723 | 0.28% [0.08, 1.00] | 1.25% [1.24, 1.26] | **0.28% [0.08, 1.00]** | 1.45% [1.44, 1.46] | 1.52% [0.85, 2.70] | 5.95% [5.92, 5.97] |
| * Adjusted for age, sex, ethnic group, residence setting, comorbidities, hospital frailty risk score, type of admission, number of previous fixation procedures and year of surgery | | | | | | | |
