# Supplementary material for: All-cause mortality following internal fixation for isolated fractures in non-polytrauma admissions: a comprehensive 10-year nationwide linked data retrospective cohort study
Source: BMJ Open. 2026 Sep 8;16(9):e122170. doi: 10.1136/bmjopen-2026-122170 (PMC13560970; doi:10.1136/bmjopen-2026-122170)
Supplement: Supplementary file 10 [file bmjopen-16-9-s008.docx]

Table H.- Crude and adjusted post-operative mortality rates by procedure

| **Procedure** | **Fracture region** | **Admissions** | **Within 30 days** | | **Within 90 days** | | **Within 365 days** | |
| --- | --- | --- | --- | --- | --- | --- | --- | --- |
|  |  |  | **Crude** | **Adjusted*** | **Crude** | **Adjusted*** | **Crude** | **Adjusted*** |
| Primary open reduction | Overall | 331,994 | **1.00% [0.97, 1.04]** | 2.10% [2.10, 2.12] | **2.10% [2.05, 2.15]** | 4.35% [4.33, 4.38] | **4.34% [4.27, 4.41]** | 8.41% [8.38, 8.45] |
|  | Shoulder girdle | 29,396 | 0.02% [0.01, 0.04] | 0.27% [0.27, 0.28] | 0.09% [0.06, 0.13] | 1.39% [1.38, 1.39] | 0.37% [0.31, 0.45] | 4.19% [4.17, 4.21] |
|  | Humerus | 34,576 | **0.35% [0.29, 0.42]** | 1.13% [1.13, 1.14] | **0.91% [0.82, 1.02]** | 3.02% [3.00, 3.03] | **2.74% [2.57, 2.92]** | 7.00% [6.97, 7.02] |
|  | Radius | 61,450 | 0.06% [0.04, 0.08] | 0.36% [0.36, 0.37] | 0.18% [0.15, 0.21] | 1.14% [1.13, 1.14] | **0.69% [0.63, 0.76]** | 3.83% [3.82, 3.85] |
|  | Ulna | 21,891 | 0.17% [0.12, 0.23] | 0.60% [0.59, 0.60] | 0.45% [0.37, 0.55] | 1.66% [1.65, 1.67] | 1.70% [1.54, 1.88] | 4.98% [4.95, 4.99] |
|  | Hand | 48,084 | 0.01% [0.01, 0.03] | 0.27% [0.27, 0.27] | 0.05% [0.04, 0.08] | 1.17% [1.17, 1.18] | 0.24% [0.20, 0.29] | 3.62% [3.60, 3.63] |
|  | Arm (other) | 346 | 0.00% [0.00, 1.10] | 0.99% [0.99, 1.00] | 0.29% [0.05, 1.62] | 2.10% [2.09, 2.11] | 1.73% [0.80, 3.73] | 6.03% [6.00, 6.05] |
|  | Pelvis | 1,676 | 0.84% [0.50, 1.40] | 1.20% [1.19, 1.20] | 2.45% [1.81, 3.30] | 3.41% [3.40, 3.43] | 5.01% [4.07, 6.16] | 7.12% [7.08, 7.15] |
|  | Femur | 55,013 | **5.42% [5.24, 5.62]** | 2.32% [2.31, 2.33] | **11.02% [10.76, 11.28]** | 4.73% [4.71, 4.74] | **20.86% [20.52, 21.20]** | 9.19% [9.17, 9.21] |
|  | Tibia | 32,008 | 0.24% [0.20, 0.30] | 1.23% [1.22, 1.24] | **0.57% [0.49, 0.66]** | 2.92% [2.91, 2.94] | **1.58% [1.45, 1.72]** | 6.69% [6.67, 6.73] |
|  | Fibula | 21,327 | 0.09% [0.06, 0.14] | 0.67% [0.67, 0.67] | 0.20% [0.15, 0.27] | 1.71% [1.70, 1.72] | 0.59% [0.49, 0.70] | 4.05% [4.03, 4.07] |
|  | Leg (Other) | 15,671 | 0.16% [0.11, 0.24] | 0.66% [0.65, 0.66] | 0.37% [0.29, 0.48] | 1.62% [1.61, 1.63] | 1.23% [1.06, 1.41] | 4.62% [4.61, 4.64] |
|  | Tarsus | 4,632 | 0.04% [0.01, 0.16] | 0.29% [0.29, 0.29] | 0.09% [0.03, 0.22] | 1.02% [1.01, 1.02] | 0.45% [0.30, 0.69] | 4.34% [4.32, 4.36] |
|  | Foot | 5,497 | 0.07% [0.03, 0.19] | 0.83% [0.83, 0.84] | 0.13% [0.06, 0.26] | 1.63% [1.62, 1.64] | 0.55% [0.38, 0.78] | 4.63% [4.61, 4.65] |
|  | Other | 427 | 0.23% [0.04, 1.31] | 1.25% [1.25, 1.26] | 0.23% [0.04, 1.31] | 1.42% [1.42, 1.43] | 1.64% [0.80, 3.34] | 5.76% [5.74, 5.79] |
| Secondary open reduction | Overall | 54,878 | 0.23% [0.19, 0.27] | 1.88% [1.87, 1.89] | 0.51% [0.45, 0.57] | 3.91% [3.89, 3.93] | **1.44% [1.34, 1.54]** | 8.04% [8.01, 8.07] |
|  | Shoulder girdle | 1,339 | 0.00% [0.00, 0.29] | 0.24% [0.24, 0.24] | 0.07% [0.01, 0.42] | 1.22% [1.22, 1.23] | 0.37% [0.16, 0.87] | 3.96% [3.95, 3.97] |
|  | Humerus | 3,679 | 0.16% [0.07, 0.36] | 1.00% [1.00, 1.01] | 0.54% [0.35, 0.84] | 2.68% [2.67, 2.69] | **2.15% [1.73, 2.67]** | 6.65% [6.63, 6.68] |
|  | Radius | 29,556 | 0.04% [0.02, 0.07] | 0.32% [0.32, 0.32] | 0.14% [0.11, 0.19] | 1.00% [0.99, 1.00] | 0.77% [0.68, 0.88] | 3.63% [3.61, 3.65] |
|  | Ulna | 1,585 | 0.19% [0.06, 0.56] | 0.53% [0.52, 0.53] | 0.38% [0.17, 0.82] | 1.46% [1.46, 1.47] | 0.82% [0.48, 1.40] | 4.72% [4.71, 4.75] |
|  | Hand | 4,780 | 0.00% [0.00, 0.08] | 0.24% [0.23, 0.24] | 0.00% [0.00, 0.08] | 1.03% [1.03, 1.04] | 0.13% [0.06, 0.27] | 3.42% [3.40, 3.43] |
|  | Arm (other) | 61 | 0.00% [0.00, 5.92] | 0.88% [0.87, 0.88] | 0.00% [0.00, 5.92] | 1.86% [1.85, 1.87] | 1.64% [0.29, 8.72] | 5.73% [5.70, 5.75] |
|  | Pelvis | 138 | 0.00% [0.00, 2.71] | 1.06% [1.05, 1.07] | 0.00% [0.00, 2.71] | 3.05% [3.03, 3.06] | 1.45% [0.40, 5.13] | 6.77% [6.74, 6.79] |
|  | Femur | 3,122 | 2.56% [2.06, 3.18] | 2.07% [2.06, 2.08] | 5.16% [4.43, 5.99] | 4.24% [4.22, 4.26] | **10.28% [9.26, 11.40]** | 8.78% [8.75, 8.81] |
|  | Tibia | 4,079 | 0.42% [0.26, 0.67] | 1.09% [1.09, 1.10] | 0.78% [0.56, 1.11] | 2.61% [2.59, 2.62] | 1.84% [1.47, 2.30] | 6.37% [6.35, 6.39] |
|  | Fibula | 4,934 | 0.14% [0.07, 0.29] | 0.59% [0.59, 0.59] | 0.26% [0.15, 0.45] | 1.51% [1.50, 1.52] | 0.69% [0.49, 0.96] | 3.83% [3.81, 3.84] |
|  | Leg (Other) | 785 | 0.25% [0.07, 0.92] | 0.58% [0.57, 0.58] | 0.51% [0.20, 1.30] | 1.43% [1.42, 1.44] | 2.17% [1.36, 3.44] | 4.37% [4.35, 4.40] |
|  | Tarsus | 457 | 0.00% [0.00, 0.83] | 0.26% [0.26, 0.26] | 0.00% [0.00, 0.83] | 0.89% [0.89, 0.90] | 1.53% [0.74, 3.13] | 4.10% [4.09, 4.12] |
|  | Foot | 338 | 0.00% [0.00, 1.12] | 0.74% [0.73, 0.74] | 0.00% [0.00, 1.12] | 1.44% [1.44, 1.45] | 0.00% [0.00, 1.12] | 4.39% [4.37, 4.40] |
|  | Other | 25 | 0.00% [0.00, 13.32] | 1.11% [1.10, 1.11] | 0.00% [0.00, 13.32] | 1.26% [1.25, 1.26] | 4.00% [0.71, 19.54] | 5.47% [5.44, 5.49] |
| Closed reduction | Overall | 210,608 | **4.37% [4.28, 4.46]** | 2.11% [2.10, 2.12] | **9.24% [9.12, 9.37]** | 4.48% [4.46, 4.49] | **17.48% [17.32, 17.65]** | 8.79% [8.77, 8.83] |
|  | Shoulder girdle | 319 | 0.00% [0.00, 1.19] | 0.27% [0.27, 0.28] | 0.00% [0.00, 1.19] | 1.44% [1.43, 1.45] | 0.00% [0.00, 1.19] | 4.42% [4.40, 4.44] |
|  | Humerus | 1,862 | **0.91% [0.57, 1.46]** | 1.13% [1.13, 1.14] | **2.85% [2.18, 3.70]** | 3.12% [3.10, 3.13] | **6.23% [5.22, 7.42]** | 7.35% [7.33, 7.37] |
|  | Radius | 16,668 | 0.10% [0.06, 0.16] | 0.36% [0.36, 0.37] | 0.25% [0.18, 0.33] | 1.18% [1.17, 1.19] | 1.17% [1.02, 1.34] | 4.06% [4.04, 4.07] |
|  | Ulna | 586 | 0.00% [0.00, 0.65] | 0.60% [0.59, 0.60] | 0.34% [0.09, 1.24] | 1.72% [1.71, 1.73] | 1.54% [0.81, 2.89] | 5.25% [5.22, 5.28] |
|  | Hand | 38,140 | 0.03% [0.01, 0.05] | 0.27% [0.27, 0.27] | 0.08% [0.06, 0.11] | 1.22% [1.22, 1.23] | 0.32% [0.27, 0.38] | 3.83% [3.82, 3.84] |
|  | Arm (other) | 100 | 1.00% [0.18, 5.45] | 1.00% [0.99, 1.00] | 1.00% [0.18, 5.45] | 2.17% [2.16, 2.18] | 1.00% [0.18, 5.45] | 6.35% [6.31, 6.37] |
|  | Pelvis | 303 | 0.99% [0.34, 2.87] | 1.20% [1.20, 1.21] | 1.65% [0.71, 3.80] | 3.53% [3.51, 3.55] | 4.95% [3.02, 8.01] | 7.47% [7.44, 7.51] |
|  | Femur | 139,617 | **6.53% [6.40, 6.66]** | 2.32% [2.30, 2.33] | **13.78% [13.60, 13.96]** | 4.87% [4.85, 4.90] | **25.87% [25.64, 26.10]** | 9.62% [9.58, 9.66] |
|  | Tibia | 9,964 | 0.38% [0.28, 0.52] | 1.23% [1.23, 1.24] | **0.80% [0.65, 1.00]** | 3.03% [3.01, 3.04] | **2.10% [1.83, 2.40]** | 7.04% [7.01, 7.06] |
|  | Fibula | 558 | 0.18% [0.03, 1.01] | 0.67% [0.67, 0.67] | 1.08% [0.49, 2.33] | 1.77% [1.77, 1.78] | 1.97% [1.10, 3.50] | 4.28% [4.26, 4.30] |
|  | Leg (Other) | 276 | 0.36% [0.06, 2.02] | 0.66% [0.66, 0.66] | 1.09% [0.37, 3.15] | 1.68% [1.67, 1.69] | 3.99% [2.24, 6.99] | 4.88% [4.86, 4.89] |
|  | Tarsus | 653 | 0.00% [0.00, 0.58] | 0.29% [0.29, 0.30] | 0.15% [0.03, 0.86] | 1.06% [1.05, 1.06] | 0.46% [0.16, 1.34] | 4.58% [4.55, 4.59] |
|  | Foot | 1,514 | 0.20% [0.07, 0.58] | 0.83% [0.83, 0.84] | 0.33% [0.14, 0.77] | 1.69% [1.69, 1.70] | 0.99% [0.60, 1.63] | 4.88% [4.86, 4.91] |
|  | Other | 48 | 0.00% [0.00, 7.41] | 1.25% [1.25, 1.26] | 0.00% [0.00, 7.41] | 1.48% [1.47, 1.49] | 0.00% [0.00, 7.41] | 6.07% [6.04, 6.09] |
| Other internal reduction | Overall | 10,849 | **0.34% [0.25, 0.47]** | 1.52% [1.51, 1.52] | **0.77% [0.62, 0.95]** | 3.40% [3.39, 3.42] | **2.18% [1.93, 2.48]** | 7.80% [7.77, 7.83] |
|  | Shoulder girdle | 415 | 0.00% [0.00, 0.92] | 0.19% [0.19, 0.19] | 0.00% [0.00, 0.92] | 1.04% [1.03, 1.05] | 0.24% [0.04, 1.35] | 3.82% [3.80, 3.83] |
|  | Humerus | 406 | **0.49% [0.14, 1.78]** | 0.80% [0.80, 0.80] | **0.49% [0.14, 1.78]** | 2.31% [2.30, 2.32] | **3.45% [2.06, 5.70]** | 6.44% [6.41, 6.46] |
|  | Radius | 321 | 0.31% [0.06, 1.74] | 0.25% [0.25, 0.25] | 0.62% [0.17, 2.24] | 0.85% [0.84, 0.85] | 1.56% [0.67, 3.59] | 3.49% [3.48, 3.50] |
|  | Ulna | 281 | 0.00% [0.00, 1.35] | 0.42% [0.41, 0.42] | 0.36% [0.06, 1.99] | 1.25% [1.24, 1.25] | 1.07% [0.36, 3.09] | 4.55% [4.52, 4.57] |
|  | Hand | 3,130 | 0.00% [0.00, 0.12] | 0.19% [0.18, 0.19] | 0.19% [0.09, 0.42] | 0.88% [0.87, 0.88] | 0.38% [0.22, 0.67] | 3.29% [3.28, 3.31] |
|  | Arm (other) | 121 | 0.00% [0.00, 3.08] | 0.70% [0.69, 0.70] | 0.00% [0.00, 3.08] | 1.59% [1.58, 1.59] | 0.00% [0.00, 3.08] | 5.53% [5.50, 5.54] |
|  | Pelvis | 126 | 0.00% [0.00, 2.96] | 0.85% [0.84, 0.85] | 0.00% [0.00, 2.96] | 2.63% [2.62, 2.64] | 0.79% [0.14, 4.36] | 6.55% [6.52, 6.58] |
|  | Femur | 2,378 | **1.22% [0.85, 1.75]** | 1.66% [1.65, 1.67] | **2.57% [2.00, 3.28]** | 3.69% [3.67, 3.71] | **6.94% [5.99, 8.03]** | 8.51% [8.48, 8.55] |
|  | Tibia | 1,169 | 0.09% [0.02, 0.48] | 0.87% [0.86, 0.87] | **0.34% [0.13, 0.88]** | 2.24% [2.23, 2.25] | **1.11% [0.65, 1.89]** | 6.16% [6.13, 6.18] |
|  | Fibula | 231 | 0.00% [0.00, 1.64] | 0.47% [0.46, 0.47] | 0.00% [0.00, 1.64] | 1.29% [1.28, 1.30] | 0.87% [0.24, 3.10] | 3.69% [3.67, 3.70] |
|  | Leg (Other) | 307 | 0.65% [0.18, 2.34] | 0.46% [0.45, 0.46] | 0.98% [0.33, 2.83] | 1.22% [1.21, 1.23] | 2.28% [1.11, 4.63] | 4.21% [4.19, 4.24] |
|  | Tarsus | 587 | 0.00% [0.00, 0.65] | 0.20% [0.20, 0.20] | 0.17% [0.03, 0.96] | 0.76% [0.75, 0.76] | 0.68% [0.27, 1.74] | 3.96% [3.94, 3.97] |
|  | Foot | 1,154 | 0.09% [0.02, 0.49] | 0.58% [0.58, 0.59] | 0.17% [0.05, 0.63] | 1.23% [1.22, 1.23] | 0.61% [0.29, 1.25] | 4.23% [4.22, 4.25] |
|  | Other | 223 | 0.45% [0.08, 2.50] | 0.88% [0.88, 0.89] | 0.45% [0.08, 2.50] | 1.07% [1.06, 1.08] | 1.35% [0.46, 3.88] | 5.28% [5.26, 5.31] |
| * Adjusted for age, sex, ethnic group, residence setting, comorbidities, hospital frailty risk score, type of admission, number of previous fixation procedures and year of surgery | | | | | | | | |
